# Opposite effects of the triple target (DNA-PK/PI3K/mTOR) inhibitor PI-103 on the radiation sensitivity of glioblastoma cell lines proficient and deficient in DNA-PKcs

**DOI:** 10.1186/s12885-021-08930-1

**Published:** 2021-11-11

**Authors:** Cholpon S. Djuzenova, Thomas Fischer, Astrid Katzer, Dmitri Sisario, Tessa Korsa, Gudrun Steussloff, Vladimir L. Sukhorukov, Michael Flentje

**Affiliations:** 1 Department of Radiation Oncology, University Hospital of Würzburg, Josef-Schneider-Strasse 11, 97080 Würzburg, Germany; 2 Department of Biotechnology and Biophysics, University of Würzburg, Würzburg, Germany

**Keywords:** DNA damage, DNA-PK, Histone γH2AX, p53, Radiation sensitivity

## Abstract

**Background:**

Radiotherapy is routinely used to combat glioblastoma (GBM). However, the treatment efficacy is often limited by the radioresistance of GBM cells.

**Methods:**

Two GBM lines MO59K and MO59J, differing in intrinsic radiosensitivity and mutational status of *DNA-PK* and *ATM*, were analyzed regarding their response to DNA-PK/PI3K/mTOR inhibition by PI-103 in combination with radiation. To this end we assessed colony-forming ability, induction and repair of DNA damage by γH2AX and 53BP1, expression of marker proteins, including those belonging to NHEJ and HR repair pathways, degree of apoptosis, autophagy, and cell cycle alterations.

**Results:**

We found that PI-103 radiosensitized MO59K cells but, surprisingly, it induced radiation resistance in MO59J cells. Treatment of MO59K cells with PI-103 lead to protraction of the DNA damage repair as compared to drug-free irradiated cells. In PI-103-treated and irradiated MO59J cells the foci numbers of both proteins was higher than in the drug-free samples, but a large portion of DNA damage was quickly repaired. Another cell line-specific difference includes diminished expression of p53 in MO59J cells, which was further reduced by PI-103. Additionally, PI-103-treated MO59K cells exhibited an increased expression of the apoptosis marker cleaved PARP and increased subG1 fraction. Moreover, irradiation induced a strong G2 arrest in MO59J cells (~ 80% vs. ~ 50% in MO59K), which was, however, partially reduced in the presence of PI-103. In contrast, treatment with PI-103 increased the G2 fraction in irradiated MO59K cells.

**Conclusions:**

The triple-target inhibitor PI-103 exerted radiosensitization on MO59K cells, but, unexpectedly, caused radioresistance in the MO59J line, lacking DNA-PK. The difference is most likely due to low expression of the DNA-PK substrate p53 in MO59J cells, which was further reduced by PI-103. This led to less apoptosis as compared to drug-free MO59J cells and enhanced survival via partially abolished cell-cycle arrest. The findings suggest that the lack of DNA-PK-dependent NHEJ in MO59J line might be compensated by DNA-PK independent DSB repair via a yet unknown mechanism.

**Supplementary Information:**

The online version contains supplementary material available at 10.1186/s12885-021-08930-1.

## Background

Radiation therapy (RT) constitutes an important approach to treating local and regional cancer. About 50–70% of all tumor patients receive RT during treatment. Tremendous advances in physical targeting and tumor imaging [1, 2] and optimization of ionizing radiation (IR) treatment protocols have yielded significant advances in patient outcome. Yet, radioresistance of tumor cells remains a major cause of treatment failure, resulting in a lower progression-free survival rate in many types of cancers, including glioblastoma (GBM), pancreatic and lung cancers. Particularly, the success rate of curing GBM remains very low with only about 10% of patients alive after 5 years following radiochemotherapy treatments [3].

In order to enhance the effects of RT, two major approaches have been proposed. The first strategy is based on the reduction of the treatment volume, i.e. by irradiating a smaller volume of healthy tissue while irradiating the defined tumor volume. The second approach utilizes the increase of the differential response of the normal tissue and the tumor using chemotherapeutics, biological agents, proteomic or genetic methods etc. [4–7]. This strategy is based on the rationale that additional artificially induced DNA damage may lower the threshold of the IR mediated cancer cell death. DNA double-strand breaks (DSBs) are the most significant lesions produced by IR and other exogenous cytotoxic agents. DSBs affect the genomic integrity of cells and, if insufficiently repaired or misrepaired, they may lead to chromosome breaks, gene deletions and translocations [8].

IR targets not only DNA itself but rather DNA in the context of chromatin, i.e. within a highly regulated and complex DNA-protein structure [9, 10]. One of the earliest events in the response to IR-induced DNA damage is phosphorylation of histone H2AX at Serin139 to γH2AX, which in turn is believed to recruit DNA repair factors to sites of DSBs [11]. Since γH2AX is associated with DSB repair, the kinetics of induction and decay of γH2AX expression might be related to the repair efficiency of higher-order chromatin structure [12]. High expression of constitutive γH2AX is indicative of defective DNA repair pathway and/or genomic instability, whereas another well-known marker protein of DNA damage, 53BP1 (p53-binding protein 1), is a conserved checkpoint protein with properties of a DNA DSBs sensor [13]. DNA DSBs attract 53BP1 to the surrounding chromatin, where 53BP1 is recruited by methylated histone H3 Lys 79 and signals chromatin/DNA damage [13].

Despite technical advances in radiation delivery protocols, poor radiation response or even radioresistance of some tumor entities justifies the urgent need to investigate the underlying network of signaling pathways that define tumor response to IR. Among others signaling pathways, the frequent activation of the PI3K pathway in GBM cells and its crucial role in cell growth, survival and migration make it a promising target for pharmacological intervention (for review, *see* [14]). Besides this, genetic alterations leading to activation of the PI3K/Akt/mTOR pathway are associated with treatment resistance in a variety of solid tumors [15].

We here examine two GBM cell lines MO59K and MO59J derived from the same glioblastoma biopsy specimen regarding their response to irradiation and simultaneous DNA-PK/PI3K/mTOR inhibition by PI-103. The two cell lines were chosen because they differ considerably in intrinsic radiosensitivity and mutational status of *PRKDC*, the gene encoding for DNA-PK_cs_. The inhibitor PI-103 was added 3 h before IR and kept 24 h thereafter. Irradiated samples of both cell lines treated with inhibitor were analyzed for colony-forming ability, interaction of tumor cells with the extracellular matrix (ECM), combination index (CI) of IR and drug treatments, DNA damage induction and repair by counting of γH2AX and 53BP1 foci, apoptosis, autophagy, marker protein expression, and cell cycle alterations.

## Methods

### Cell lines and preliminary characterization

Glioblastoma cell lines MO59K and MO59J were obtained from the American Type Culture Collection (Manassas, VA) and authenticated by the supplier. Both cell lines were cultivated in CGM containing Dulbecco’s modified Eagle’s medium nutrient mixture F-12 HAM (Sigma D-6421), non-essential amino acids, and sodium pyruvate according to the provider’s prescription. The modal chromosome numbers were reported to be 64 and 74 for MO59K and MO59J lines, respectively [16]. Population doubling times were found to be 60 ±2 and 72 ±3 hrs for the MO59K and MO59J cell lines, respectively. Cells were used at low (< 15) passages after thawing and were regularly examined during the study for Mycoplasma (MycoAlert; Lonza, Rockland, ME) contamination. Both cell lines are mutated in *p53* and *PTEN* genes [https://web.expasy.org/cellosaurus/CVCL_0401;https://web.expasy.org/cellosaurus/CVCL_0400) [17]].

In our preliminary tests (*see* Additional file 1: Supplemental Methods, Additional file 1: Figs. S1-S4) we found intrinsic differences in chromatin compaction between the two tested cell lines. These tests were performed because chromatin compaction is well known to play an essential role in DNA repair, and thus can influence the sensitivity of cells to DNA damaging agents [18]. To this end, we performed a Comet assay and also analyzed microscopically the expression of histone deacetylase 5 (HDAC5). Additionally, we tested the stainability of the nuclear chromatin with 4′,6′-diamidino-2-phenylindole (DAPI).

The alkaline Comet assay reveals not only single-strand DNA breaks and alkali-labile sites, but it also provides a measure for chromatin integrity after exposure to a clastogenic agent such as IR [19]. By assessing the DNA damage/fragmentation from the Tail Moment (TM) values of irradiated cells (Additional file 1: Fig. S1), we found that the initial radiation-induced DNA fragmentation in MO59J cells was ~ 1.3 times higher than in MO59K cells. Yet, the residual DNA damage as well as the DNA repair kinetics were very similar in both cell lines. The difference between MO59K and MO59J cell lines was seen more evidently if the TM distribution of individual cells was taken into account, i.e. the representative TM values histograms of the radiosensitive MO59J line were strongly shifted towards higher values compared with the TM values histograms of MO59K cells (Additional file 1: Fig. S2).

A further preliminary test was immunofluorescence staining of HDAC5 foci. It is known that histone deacetylation promotes a “closed” chromatin conformation and it usually leads to repression of gene activity [20]. As seen in Additional file 1: Fig. S3, the number of HDAC5 foci in MO59K cells was ~ 10-time higher than in MO59J cells. An additional interesting finding was that MO59K cells show a much higher stainability with DAPI than MO59J cells (Additional file 1: Fig. S4). Since a linear relationship exists between chromatin condensation and its stainability with DAPI [21], this result points to a strong intrinsic difference in chromatin compactness between the two cell lines studied here. The finding on DAPI staining (Additional file 1: Fig. S4) also corroborates with the observed difference in HDAC5 expression (Additional file 1: Fig. S3). To sum up, the preliminary tests, including the Comet assay, HDAC5 and DAPI staining, suggest a strong difference in the intrinsic degree of chromatin condensation between the MO59K and MO59J cell lines.

### Drug treatment and cytotoxicity of PI-103

The inhibitor PI-103 was purchased from Selleckchem (Absource Diagnostics GmbH, Munich, Germany). The substance was freshly diluted from frozen (− 80 °C) aliquots dissolved in DMSO. The cytotoxicity of PI-103 within the concentration range 0.01–20 μM was studied by an ATP-based assay (CellTiter-Glo Luminescent Cell Viability Assay, Promega, Madison, WI) according provider’s prescription. The cellular ATP levels in cell samples treated with the drug for 24 h were normalized against DMSO-treated controls and plotted versus PI-103 concentration (Additional file 1: Fig. S5). With increasing PI-103 concentration, the mean ATP content in both cell lines decreased steadily depending on the cell line to about 20–40% of the initial level after drug (20 μM) exposure. Based on these measurements, 2 μM of PI-103, causing about 15–20% viability loss, was used for subsequent experiments. The selected PI-103 concentration is consistent with the previously reported data [22]. PI-103 (2 μM, [22]) was added 3 h (short-term treatment) prior to exposure to IR and remained in CGM up to 24 h (long-term treatment) after IR. Control cells were treated in parallel with respective amounts of DMSO.

#### X-ray irradiation

Irradiation was performed at room temperature using a 6 MV Siemens linear accelerator (Siemens, Concord, CA) at a dose rate of 2 Gy/min. After irradiation, cells were kept in CGM for the indicated time until harvest.

#### Colony survival assay

Cell survival was assessed by colony formation as previously described [22]. Subconfluent monolayers of control and inhibitor-treated cells were irradiated in culture flasks filled with CGM at room temperature by graded single doses (0–8 Gy), seeded 24 h post-IR in Petri dishes, i.e. delayed plating, and then cultured for 10–12 days in CGM. Four replicates were performed for each radiation dose, and the experiments were repeated at least four times. After 10–12 days the cells were fixed and stained with crystal violet (0.6%). Macroscopic colonies containing at least 50 cells were scored as survivors. The mean clonogenic survival data for each cell line were fitted to the linear-quadratic (LQ) model:

1 $$ \mathbf{SF}=\mathbf{\exp}\left(\hbox{-} \boldsymbol{\upalpha} \mathbf{X}\hbox{-} {\boldsymbol{\upbeta} \mathbf{X}}^{\mathbf{2}}\right) $$

where, *SF* is the survival fraction, *X* is the irradiation dose, α and β are the fitted parameters.

In some experiments, radiation survival was assessed without replating, i.e. tumor cells were cultured at fixed densities, and treated with the inhibitor 3 h before IR with 3 and 5 Gy. Twenty four hours after IR culture medium was replaced with fresh medium, and the dishes were incubated for the next 10–12 days, fixed and stained as it was done in case of delayed plating (*see* above).

To determine the type of combination effect of X-ray with PI-103 in MO59K cells, the CI value was calculated as previously described [23, 24]. To this end, MO59K cells were treated with different doses (2, 3 and 5 Gy) of IR, or different concentrations of PI-103 (0.5, 1 and 2 μM) or a combination of radiation (3 Gy) with different concentrations of PI-103. The treated cell samples were then examined for colony-forming ability as described above. The CI is a quantitative measure of the degree of interaction between different treatments. CI values in the range of 0.9–1.1 indicate additive effect, whereas values greater than 1.1 indicate antagonism. CI values in the range from 0.9 to 0.7 indicate slight synergism and CI values less than 0.7 synergism.

To study extracellular matrix-dependent colony formation, the Petri dishes were pre-coated with either fibronectin (FN) or bovine serum albumin (BSA) as previously described [25]. Clonogenic survival of non-irradiated and irradiated control and drug-treated cells was performed as explained above without re-plating.

#### Western blotting

For immunoblot assays, whole-cell lysates were prepared 30 min and 24 h post-IR, according to standard procedures. Samples equivalent to 40 μg of protein were separated using 4–12% SDS-polyacrylamide pre-cast gels (Invitrogen, Karlsruhe, Germany) and transferred to nitrocellulose membranes. For protein detection, membranes were incubated with respective primary and species-specific peroxidase-labeled secondary antibodies according to standard protocols. The levels of protein expression were quantified using the software ImageJ (NIH, Bethesda, MD) and normalized to β-actin levels.

#### Antibodies

The primary and secondary antibodies are specified in the Additional file 1: Supplemental Materials.

#### γH2AX and 53BP1 foci counting using confocal laser scanning microscopy (CLSM)

Cells (about 5·10^4^) were seeded in 500 μl medium per well in 8-well Chamber Slides (Sarstedt, Nümbrecht, Germany) 20–24 h before irradiation. Cells were treated with PI-103 for 3 h prior to radiation with a single dose of 2 Gy. After irradiation, cells were kept in CGM for the indicated time until fixation. At different time points (0 min, 10 min, 20 min, 30 min, 120 min, 240 min and 360 min) after irradiation, cells were washed with pre-warmed (37 °C) PBS and fixed for 10 min at room temperature with a PBS solution containing 4% formaldehyde (Thermo Scientific, Rockford, IL). Thereafter, the cells were stained simultaneously for γH2AX and 53BP1 as previously decribed [26, 27]. Confocal fluorescence images were acquired with a Zeiss LSM 700 microscope. In each sample, computer-assisted γH2AX foci counting from 3D-CLSM image stacks [28] was performed in about 100 cells.

#### Cell cycle measurements by flow cytometry

Non-treated and drug-treated cell cultures were irradiated as sub-confluent monolayers in CGM at room temperature. The cells were then kept in CGM under standard conditions, and analyzed by flow cytometry 30 min and 24 h after IR exposure. For analysis, the cells were trypsinized, washed twice in PBS, fixed and stained with propidium iodide (PI, Sigma P-4170, 10 μg ml^− 1^) in the presence of ribonuclease A (Sigma R-5250, 25 μg ml^− 1^) as described elsewhere [22].

At least 20,000 cells were assayed for DNA distribution using a flow cytometer FACSCantoII (Becton Dickinson, San Jose, CA). The output data presented as one-dimensional histograms acquired in linear mode, i.e., the distributions of PI-DNA signals within the cell samples, were analyzed using the Flowing Software program obtained from P. Terho (Turku Centre for Biotechnology, Turku, Finland) and the ModFit LT program (Verity Software House, Topsham, ME).

#### Statistics

Data are presented as means (± SD or ± SE). Mean values were compared via Student’s *t-*test. The threshold of statistical significance was set at *P* < 0.05. Statistics and fitting of experimental data were performed with Origin 8.5 (Microcal, Northampton, MA).

## Results

The following experiments were performed to evaluate the effects of simultaneous targeting of DNA-PK, PI3K and mTOR with PI-103 on the radiation sensitivity, marker protein expression, DNA damage/repair, the degree of apoptosis, autophagy, and cell cycle alterations in two GBM tumor cell lines. The two commercially available (MO59K and MO59J) cell lines originate from different portions of the same human glioblastoma specimen and show an ~ 10-fold difference in their radiation sensitivities [29]. In addition, the radiosensitive MO59J cells carry mutated *DNA-PKcs* and *ATM* genes [30]. Given that MO59J cells are fully devoid of DNA-PKcs, the pyridinylfuranopyrimidine PI-103, a triple-target (DNA-PK, PI3K and mTOR) inhibitor, has one less target in MO59J cells, as compared to MO59K cells, which express all three target proteins.

### Effects of PI-103 and NVP-AUY922 on colony survival after IR

**Fig. 1:**
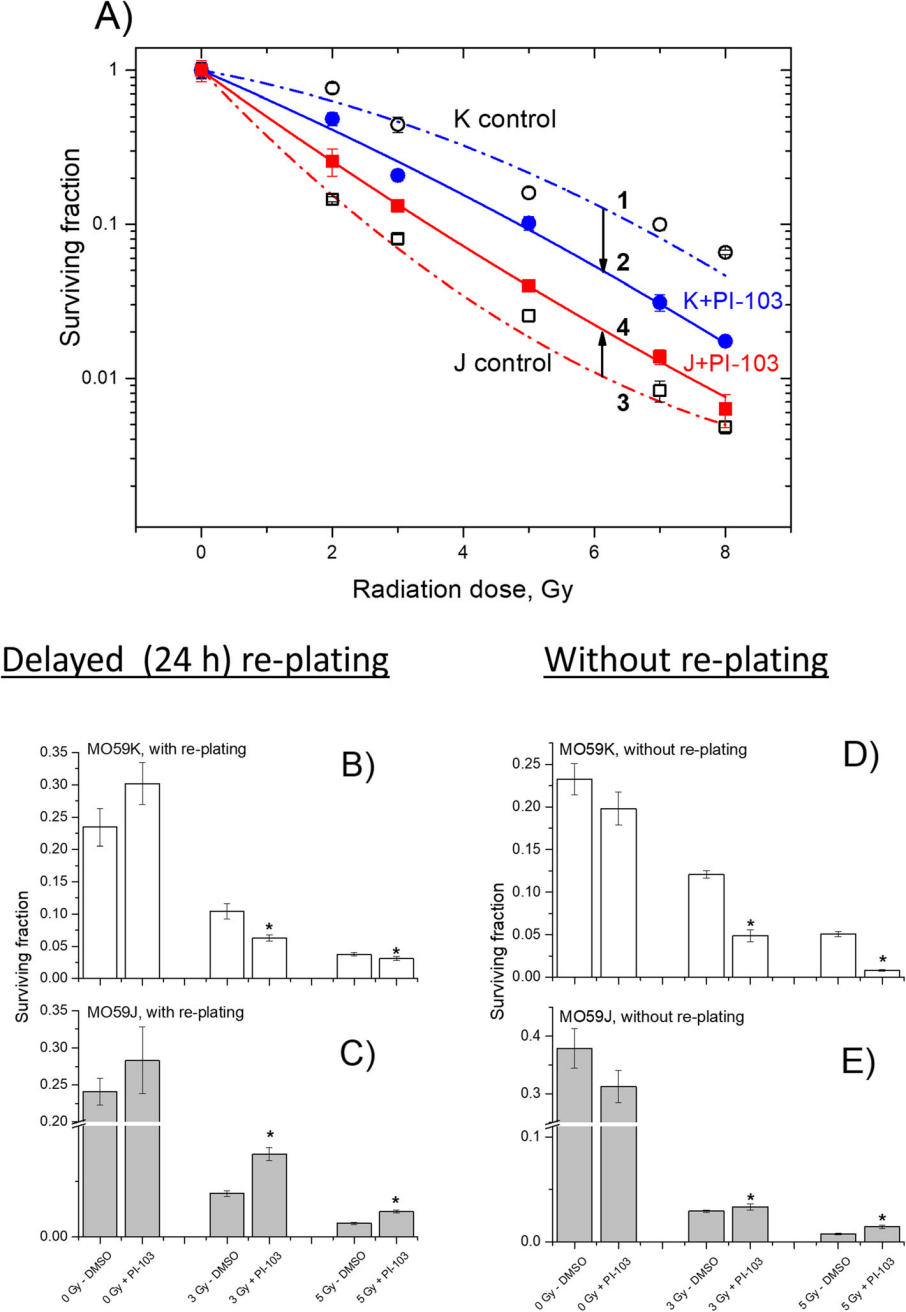
Clonogenic survival of MO59K and MO59J tumor cell lines treated with PI-103 for 3 h prior to IR. Irradiated cells were either re-plated for the colony-forming test 24 h after IR (**a**, **b**, **c**) or were seeded at fixed cell densities, drug treated, irradiated, and 24 h after IR drug-containing medium was replaced with fresh medium (**d**, **e**). After 10–12 days, colonies containing at least 50 cells were scored as survivors. Data derived from at least three independent experiments for each cell line were pooled together (**a**) and fitted by a linear-quadratic equation (Eq. 1). The SD values are indicated by error bars. Parts **b** and **c** show the survival data at 3 and 5 Gy extracted from part **a**. Parts **d** and **e** showed the survival data at 3 and 5 Gy obtained without re-plating. “*” means *P* < 0.05

Figure 1 shows the cell survival curves of control (DMSO) and drug-treated cells plotted versus the radiation dose, along with the best fits of the LQ model (Eq. 1) to the data. The plating efficiencies (PE) of non-irradiated cell samples, as well as the fitted parameters derived with the LQ model, including the surviving fraction at 2 Gy (SF2), the radiation dose required to reduce colony forming ability by 90% (D_10_) and the growth inhibition factor (IF_10_) from at least 4 independent experiments are summarized in the Additional file 1: Table S1. As seen in Fig. 1a, PI-103 had a strong radiosensitizing effect in MO59K cells (Fig. 1, curve 2 vs. curve 1), as evidenced by the decrease of the SF2 value from 0.63 in non-treated irradiated cells to 0.41 in PI-103-treated irradiated MO59K cells (Additional file 1: Table S1). In contrast, PI-103 induced radioresistance in MO59J cells (Fig. 1, curve 4 vs. curve 3), indicated by the increase of the SF2 value from 0.16 in control irradiated MO59J cells to 0.23 (Additional file 1: Table S1). Likewise, the D_10_ value was increased from 2.3 Gy in control to 3.2 Gy in drug-treated MO59J cells.

It is conceivable that radiation resistance induced by PI-103 in MO59J cells may be the result of selective cell loss prior to cell re-plating due to excessive apoptosis and increased detachment of highly damaged cells treated with PI-103 and IR. These cells would escape analysis due to the washing and trypsinization steps prior to subsequent re-plating for colony-survival test. Indeed, the colony-survival test in our study was carried out with delayed plating, i.e. the cell samples were incubated 24 h post-IR with the inhibitors before being re-plated in Petri dishes for further cultivation. In order to analyze the possibility of exclusion of strongly damaged cells during delayed plating, we tested additionally the survival of *pre-*plated cells. To this end, the tested cells were grown at fixed densities in Petri dishes, treated with the inhibitor for 3 h and irradiated with a single dose of 3 and 5 Gy. Twenty four hours later the inhibitor was washed out and the samples were cultivated just like in the delayed plating protocol. The results of experiments without re-plating are shown in Fig. 1d, e. As seen in Fig. 1d, e, treatment with PI-103 without re-plating caused the same opposing effects on the radiation sensitivity of the two cell lines as in the experiments with delayed re-plating (Fig. 1b, c), i.e. diminished (MO59K) and enhanced (MO59J) survival of irradiated PI-103-treated cells. Moreover, PI-103 acts much stronger as a radiosensitizer in MO59K cells treated under the protocol without re-plating (Fig. 1d) compared to the experiments with delayed re-plating (Fig. 1b).

The type of combination effect of IR and PI-103 on MO59K cells was determined by calculating the CI value [23, 24]. In these experiments, MO59K cells were treated either with different doses of radiation (Additional file 1: Fig. S6a), or with PI-103 (Additional file 1: Fig. S6b) or with a combination of radiation (3 Gy) and PI-103 (Additional file 1: Fig. S6c) added 3 h before IR, and clonogenic survival of MO59K cells was determined as described in the section Materials and Methods. The experiments revealed that the interactions of radiation and PI-103 were mostly synergistic (CI < 0.7), which suggest a radiosensitizing effect of PI-103 on MO59K cells.

Another important aspect of radiation sensitivity is the interaction of cancer cells with the extracellular matrix (ECM). The presence of an ECM increases cellular resistance to cell-damaging agents such as drugs or IR [25]. To test whether FN or BSA influences the clonogenic survival of MO59K and MO59J cells, Petri dishes were coated [25] with either FN or BSA according to Cordes et al. (2003). The results (Additional file 1: Fig. S7) showed that pre-coating of dishes with FN or BSA had no impact on the clonogenic survial of control and drug-treated and/or irradiated cells of both cell lines.

To gain additional insight into the interaction of cancer cells with the extracellular matrix and to interpret the results in Fig. S7, we studied the expression of several marker proteins involved in cell adhesion. These were focal adhesion kinase (FAK), p-FAK (Ser910), integrin-linked kinase (ILK1), RhoA, cdc42 and Rac1/2/3 proteins (Additional file 1: Fig. S8). As seen in Fig. S8, the background expression levels of the analyzed proteins were very similar in both cell lines, and neither radiation nor PI-103 significantly affected the expression of the studied proteins.

### Effects of PI-103 and irradiation on the expression of marker proteins of the PI3K- and MAPK-pathways

**Fig. 2:**
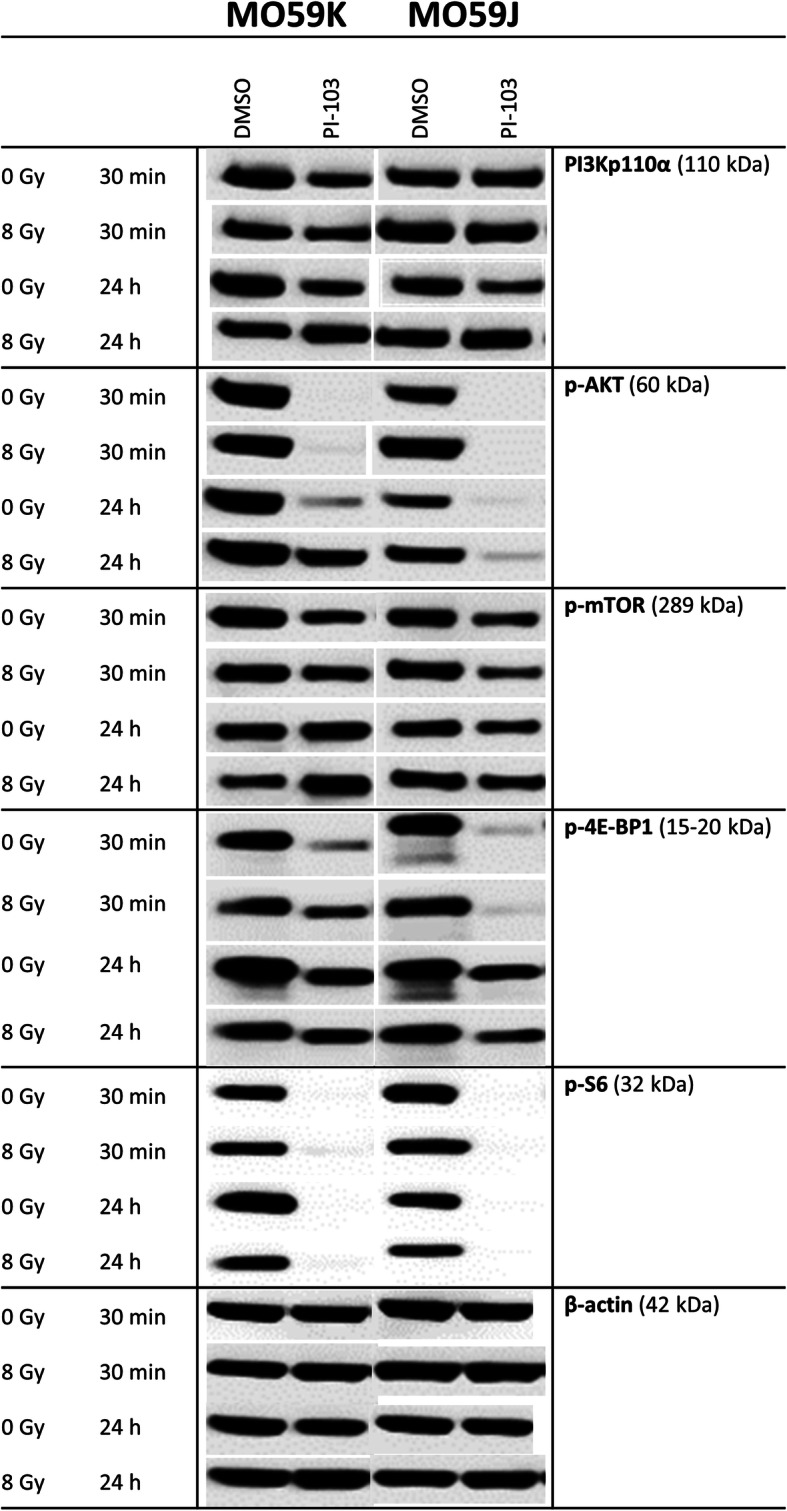
Representative Western blot analysis of expression levels of several marker proteins of PI3K-pathway in MO59K and MO59J tumor cells treated with DMSO (control) or PI-103 for 3 h prior to IR with 8 Gy and detected 30 min and 24 h thereafter. β-actin was used as loading control. The uncropped blots are shown in Fig. S12. The experiment was repeated at least three times

In order to explain the opposing effect of the triple inhibitor PI-103 on the radiation sensitivity of MO59K and MO59J cells we analyzed the expression of two groups of proteins. The first group (Fig. 2) includes several marker proteins of the PI3K-pathway, i.e. PI3K, p-Akt (Ser473) and p-mTOR, along with p-4E-BP1 and p-S6. The second group includes three proteins of the MAPK-pathway, i.e. Raf-1, p-MEK1/2 and p-Erk1/2 (Additional file 1: Fig. S6). Figure 2 and Fig. S6 show exemplarily the Western blot data of control and drug-treated samples of both cell lines probed for the marker proteins in control, drug-treated and/or irradiated cell samples. Samples shown on the left- and right-hand sides (LHS, RHS) of Figs. 2, S6 were obtained from MO59K and MO59J cells, respectively.

As seen in Fig. 2 (LHS column), a short (3 h) incubation with PI-103 slightly decreased the expression of PI3K in MO59K cells, whereas the PI3K level in the MO59J cell line remained mostly unchanged. At the same time, the amount of p-Akt was completely depleted in PI-103-treated samples after a 3-h drug treatment in both cell lines, with and without IR. In contrast, 24-h treatment with PI-103 caused reactivation of p-Akt [22], i.e. the amount of phosphorylated form of Akt in both non-irradiated and especially in irradiated MO59K cells recovered to about 50–80% of the background level (Fig. 2, LHS). However, in PI-103-treated MO59J cells much less Akt was phosphorylated independent of IR.

In addition to PI3K, a target of PI-103, we analyzed the presence of a further target of PI-103, mTOR and its downstream effectors, ribosomal S6 and translational repressor 4E-BP1 proteins, which are known to influence cell-cycle progression and cell growth [31, 32]. The amount of functional m-TOR, p-mTOR was decreased after a 3-h incubation with PI-103 in both cell lines (Fig. 2), independent of IR. However, after a 24-h incubation it was moderately *increased* in MO59K cells whereas it remained slightly reduced in MO59J cells. As a result of depletion of p-Akt and reduction of p-mTOR, the p-S6 protein was also depleted 30 min post-IR in both cell lines (Fig. 2) treated with PI-103. In contrast, the amount of p-4E-BP1 was reduced to a much lesser extent (Fig. 2) but in the same pattern as p-S6.

The lack of *PTEN* in *PTEN*-mutated cells, such as both tested cell lines, usually leads to a compensatory activation of the PI3K pathway. Thus, activation of Akt, a major hub protein of the pathway, typically results in an inhibition of Raf-1 and its downstream effectors MEK and ERK through a cross-talk between the PI3K/Akt/mTOR and Ras/Raf/MEK/Erk (MAPK signaling) pathways [33]. Normally the MAPK pathway transmits signals from cell surface receptors to promote proliferation and survival programs, and it is frequently mutated in cancer cells [34, 35]. The expression of Raf-1 (Additional file 1: Fig. S6) remained nearly unchanged after incubation with PI-103 in both cell lines. Similarly, no significant effects of PI-103 on the amounts of p-MEK1/2 und p-Erk1/2 (Additional file 1: Fig. S6) were seen in both cell lines. Interestingly, the background expression of p-Erk1/2 was much stronger in MO59J cells compared with the MO59K line, especially in irradiated MO59J cells 30 min post-IR.

In addition, we detected the expression of non-phosphorylated forms of the above mentioned proteins (data not shown). Contrary to the phosphorylated forms, the expression of non-phosphorylated forms of Akt, mTOR, 4E-BP1, S6, MEK1/2, and Erk1/2 remained almost unchanged after addition of PI-103.

### Impact of PI-103 on the IR-induced DNA damage assessed by γH2AX and 53BP1 foci counting

**Fig. 3:**
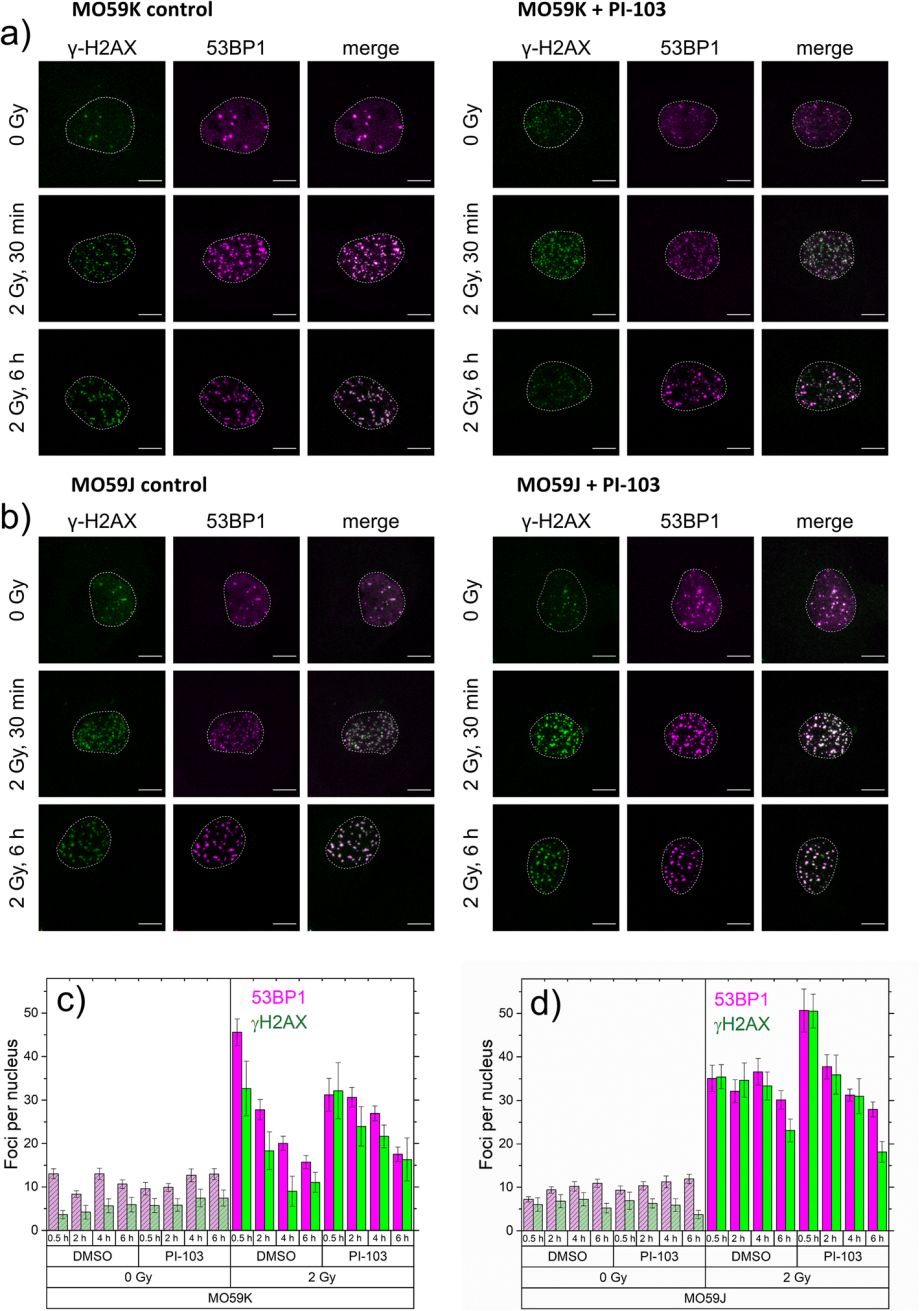
DNA damage assessed by histone γH2AX and 53BP1 foci counts per nucleus in control or drug-treated and/or irradiated M059K (**a**) and M059J (**b**) cells. Cells were treated with PI-103 3 h prior to irradiation with 2 Gy, fixed at different time points (30 min, 2 h, 4 h and 6 h) after irradiation and immunostained for γH2AX and 53BP1. DNA damage was assessed by the mean number of γH2AX (green) and 53BP1 (magenta) foci per nucleus in non-irradiated and irradiated (2 Gy) cells, either in control or in cells treated with PI-103 fixed 30 min, 2 h, 4 h and 6 h post-irradiation. For each time point at least 100 nuclei were evaluated using the ImageJ plugin FocAn [28]

To further elucidate the opposing effects of PI-103 on the colony-forming ability of MO59K and MO59J cells (Fig. 1), we compared the IR-induced DNA damage in PI-103-treated and control drug-free cells by counting immunostained γH2AX and 53BP1 foci [11, 26, 27] as markers of the DNA DSBs in irradiated cell samples (Fig. 3a, b), either untreated or pretreated with PI-103. γH2AX and 53BP1 foci counting in cell nuclei (Fig. 3c, d) was performed on 3D-CLSM image stacks using an automated foci analysis software reported recently [28]. The γH2AX and 53BP1 foci were counted in samples prepared at different times (0–6 h) after IR.

We found substantial amounts of γH2AX and 53BP1 foci not only in irradiated but also in non-irradiated samples of both GBM lines (Fig. 3). Even without being exposed to IR (Fig. 3), drug-free control samples of both cell lines displayed a base level of ~ 8–10 foci per nucleus (fpn) over the 6 h of observation time. These values are well within the range reported for spontaneous γH2AX foci caused by replication-associated breaks in a variety of cancer cell lines [26, 36]. PI-103 had little, if any, effect on the foci number in non-irradiated samples (Fig. 3).

Within the first 30 min after irradiation with 2 Gy, the amount of γH2AX and 53BP1 foci in drug-free MO59K and MO59J cells increased rapidly to comparable peak values of ~ 45 and ~ 35 fpn, respectively (Fig. 3c, d). After that, the foci numbers decreased steadily in both cell lines, however, to a different extent. The foci numbers of both proteins in MO59J cells decayed much slower than in MO59K cells. Thus, 6 h after irradiation, the residual foci numbers of both proteins in MO59K and MO59J cells were found to be, respectively, 10–15 and 20–35 fpn. The large ~ 2.5 fold excess of residual (~ 25 fpn) over spontaneous (~ 10 fpn in non-irradiated) foci numbers suggests that the IR-induced DNA DSBs in non-treated irradiated MO59J cells have been only partially repaired within 6 h after irradiation. Treatment of MO59K cells with PI-103 lead to protraction of the DNA damage repair as compared to drug-free irradiated cells (Fig. 3c). Interestingly, in PI-103-treated and irradiated MO59J cells the expression of foci of both proteins was higher than in the drug-free samples, but a large portion of DNA damage was quickly repaired (Fig. 3d).

Although the drug-free samples of MO59K and MO59J cell lines were similar in their initial γH2AX and 53BP1 foci counts (Fig. 3c, d), PI-103 exerted different effects on the time-course of foci induction and decay in two cell lines. However, for a deeper quantitative analysis of γH2AX foci in these isogenic cell lines, the ~ 10% difference in their modal chromosome numbers [16] should also be considered.

### Effects of PI-103 and irradiation on the expression of DNA repair proteins

**Fig. 4:**
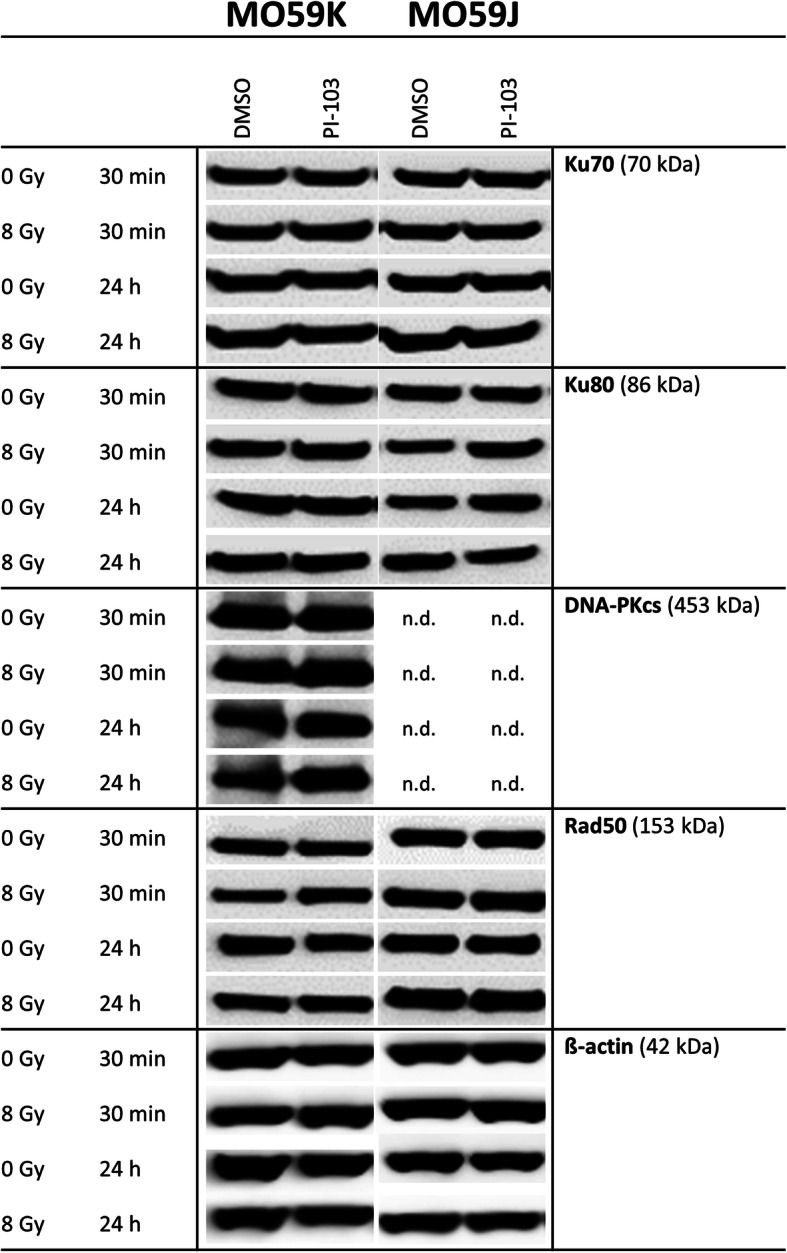
Representative Western blot analysis demonstrating the expression of several DNA repair proteins of the NHEJ pathway in MO59K and MO59J cells treated with DMSO (control) or PI-103 for 3 h prior to IR with 8 Gy and detected 30 min and 24 h thereafter. β-actin was used as loading control. The uncropped blots are shown in Fig. S12. The experiment was repeated at least three times

**Fig. 5:**
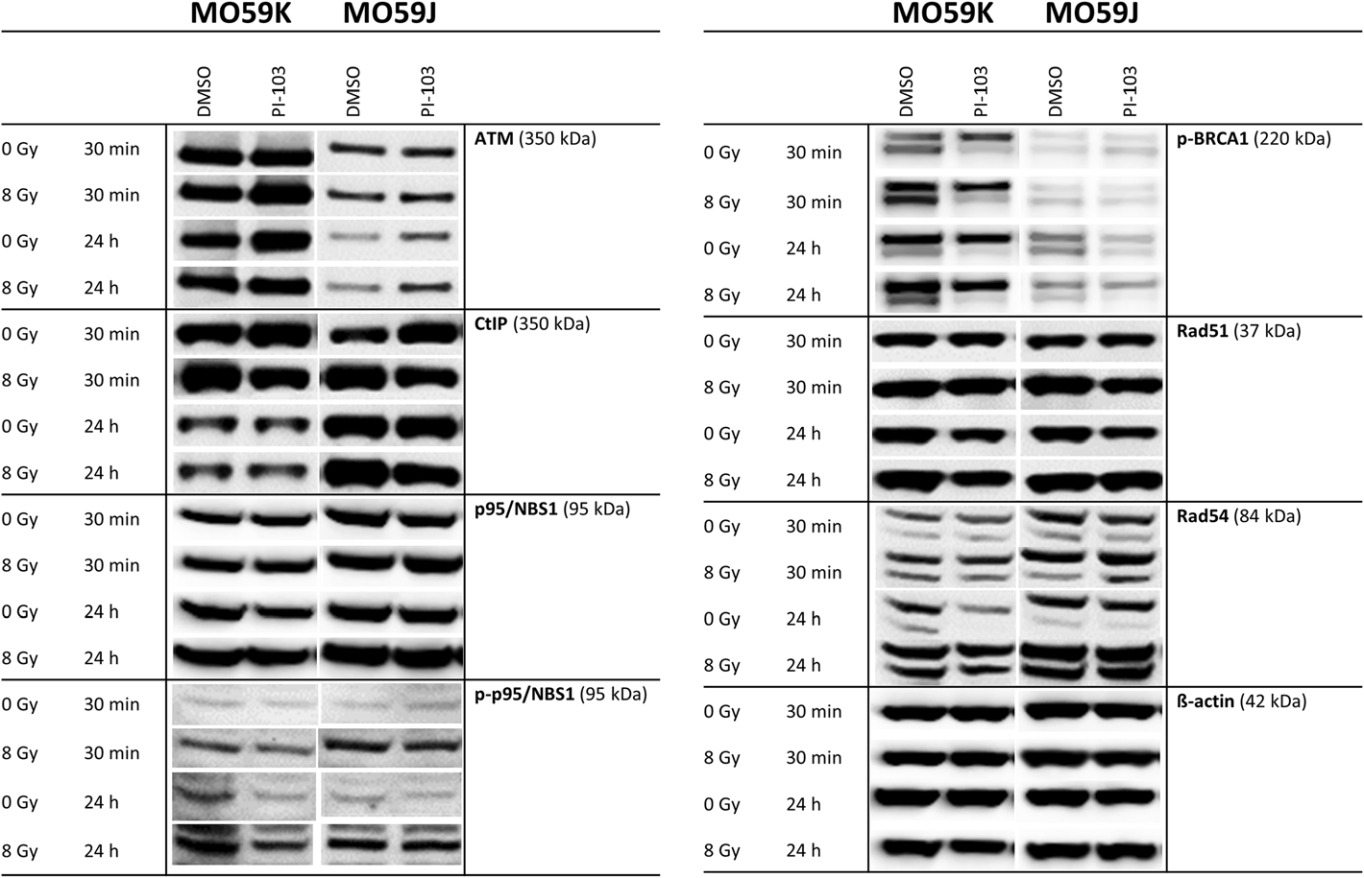
Representative Western blot analysis of the expression levels of several DNA repair proteins of the HR pathway in MO59K and MO59J cells treated with DMSO (control) or PI-103 for 3 h prior to IR with 8 Gy and detected 30 min and 24 h thereafter. β-actin was used as a loading control. The uncropped blots are shown in Fig. S12. The experiment was repeated at least three times

Driven by the finding that PI-103 inversely affects the radiation survival of tested cell lines (Fig. 1) we analyzed the expression of several DNA repair proteins. Figure 4 and Fig. 5 show representative Western blot detections of several proteins belonging either to non-homologous end-joining (NHEJ, Fig. 4) or homologous recombination (HR, Fig. 5) DNA repair pathways in both cell lines treated with drug and IR.

As seen in Fig. 4, the components of the heterodimer Ku70/Ku80, which bind to and protect the broken DNA ends, are equally expressed in both cell lines and the expression levels were unaffected by drug and/or IR treatment. The deficiency of MO59J cells in *DNA-PK* gene is clearly confirmed by our Western blot data (Fig. 4, RHS), i.e. DNA-PK was completely absent in MO59J cells but clearly present in MO59K cells (Fig. 4, LHS). Thirty minutes after IR, the expression levels of DNA-PK in MO59K cells were almost unchanged, independent of drug and/or IR treatment (Fig. 4). We also found no differences in the background expression of the DNA repair protein Rad50 between cell lines (Fig. 4). Moreover, neither chemical inhibition nor IR exposure affected Rad50 expression in both studied cell lines.

In addition to the NHEJ DNA repair pathway, we tested several proteins of the HR pathway (Fig. 5). Our Western blot data (Fig. 5, RHS) are in line with the mutation in *ATM* gene in MO59J cells, i.e. the expression of ATM protein was strongly reduced in this line compared with MO59K line. Interestingly, despite the ATM deficiency in MO59J cells, addition of PI-103 clearly increased ATM expression thereby enhancing DNA repair. We further tested the CtBP (C-terminal binding protein) interacting protein (CtIP), an interacting partner of the Mre11/Rad50/Nbs1 (MRN) DNA damage sensor protein complex, which recognizes DNA DSBs. As seen in Fig. 5, CtIP as well as p95/NBS1 are strongly expressed in both cell lines, especially 24 h post-IR in MO59J cells. In both cell lines, the activated form of p95/NBS1 protein was strongly induced by IR independent of PI-103 treatment. Additionally we detected p-BRCA1 protein, which, together with BRCA2, is required for localization of Rad51 to DNA DSBs sites. As seen in Fig. 5, MO59J cells exhibited lower amount of p-BRCA1 as compared to MO59K cells. PI-103 treatment slightly diminished p-BRCA1 content in non-irradiated MO59J cells, but not in irradiated samples. The expression pattern of Rad51 was similar to that of p-BRCA1. The Rad54 helicase, which interacts with Rad51 to regulate its DNA binding and strand exchange activities during HR, was strongly expressed in both cell lines, especially 24 h post-IR in MO59J cells independent of drug treatment.

To sum up, we found that several proteins of the NHEJ pathway are highly expressed not only in the repair-proficient MO59K cells but also in the repair-deficient MO59J cells. However, due to the absence of DNA-PK, NHEJ likely does not contribute to DSB repair upon drug and/or IR treatment in MO59J cells. As for HR, despite reduced level of ATM in MO59J cells, other marker proteins of the HR pathway were markedly expressed in this cell line, except for p-BRCA1. Interestingly, addition of PI-103 did not reduce the expression of HR-related proteins, but instead it *induced* the expression of ATM especially in MO59J cells, indicating a certain degree of HR pathway functionality in the radiation-sensitive MO59J cells.

### Effects of PI-103 and irradiation on the expression of p53, p-p53 and p53-related Bax protein

**Fig. 6:**
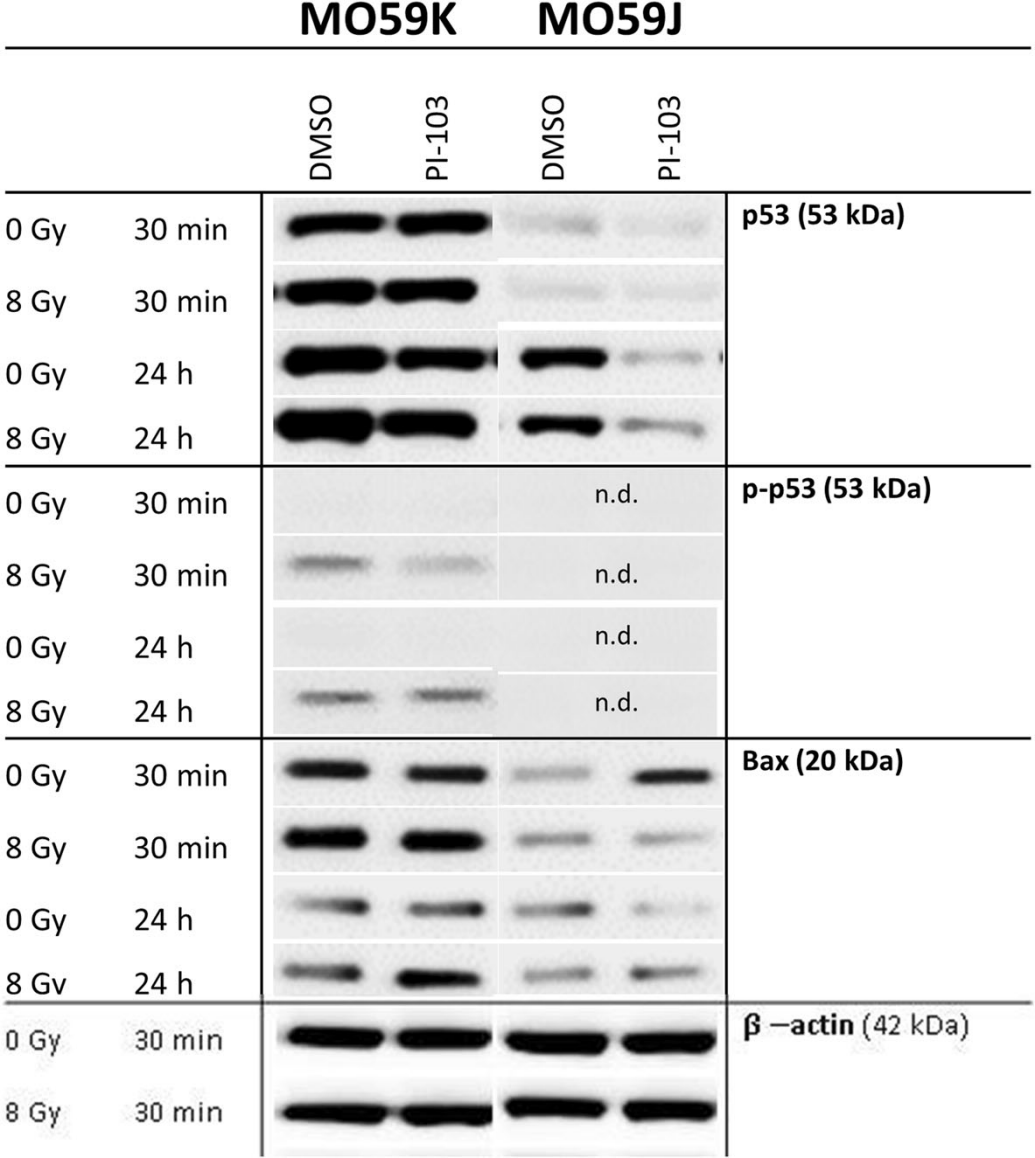
Western blot analysis of p53, p-53 and Bax proteins in MO59K and MO59J cells treated with DMSO (control) or PI-103 for 3 h prior to IR with 8 Gy and detected 30 min and 24 h thereafter. The uncropped blots are shown in Fig. S12. β-actin was used as loading control. The experiments were repeated at least three times. For details, *see* legend to Fig. 2

Both DNA-PK and ATM redundantly phosphorylate similar substrates, e.g. both are required for normal levels of p53 phosphorylation and p53-dependent apoptosis [37]. Therefore, we analyzed the expression of p53 in both cell lines (Fig. 6). We found that the diminished expression of p53 in MO59J cells was further reduced by addition of PI-103 (24 h post-IR) but to a much lesser extent in MO59K cells (Fig. 6). Another difference between these cell lines was that irradiation activated p53 in MO59K cells by phosphorylation on Serin15 (Fig. 6), but p-p53 was undetectable in irradiated MO59J cells. Next, we found that at the time of IR the background expression of the pro-apoptotic protein Bax was much higher in MO59K cells than in MO59J cells, especially after addition of PI-103 (Fig. 6). Given that Bax is related to p53, this finding is in line with the pro-apoptotic function of p53 and its different expression levels in both cell lines.

### Effects of PI-103 and radiation on late-stage apoptosis and autophagy

To further explore the mechanisms underlying the opposing effects of PI-103 on the radiation sensitivity of the GBM cell lines illustrated in Fig. 1, we also analyzed cleaved PARP, a well-known marker of apoptosis. As seen in Additional file 1: Fig. S7, the background amount of PARP, which plays an important role not only in base excision repair, but also in homologous and non-homologous DNA repair [38], was much higher in MO59K than in MO59J cells. Interestingly, short incubation with PI-103 strongly increased the presence of cleaved PARP, especially in MO59K cells (Additional file 1: Fig. S7) and this effect was independent of IR. This finding is consistent with the assumption that during IR, PI-103-treated MO59K cells underwent extensive apoptosis. The net amount of cleaved PARP in drug-treated MO59J cells was much lower than in MO59K cells, which may be due to the deficiency of MO59J cells in DNA-PK, which is normally involved in signaling DNA damage to the apoptosis machinery [39]. In contrast to short-term incubation with the inhibitor, after long-time incubation with the drug, almost no presence of cleaved PARP was seen, independent of IR. Another tested marker of apoptosis, cleaved caspase 3, did not show any changes in response to the inhibitor and/or IR (Additional file 1: Fig. S7).

**Fig. 7:**
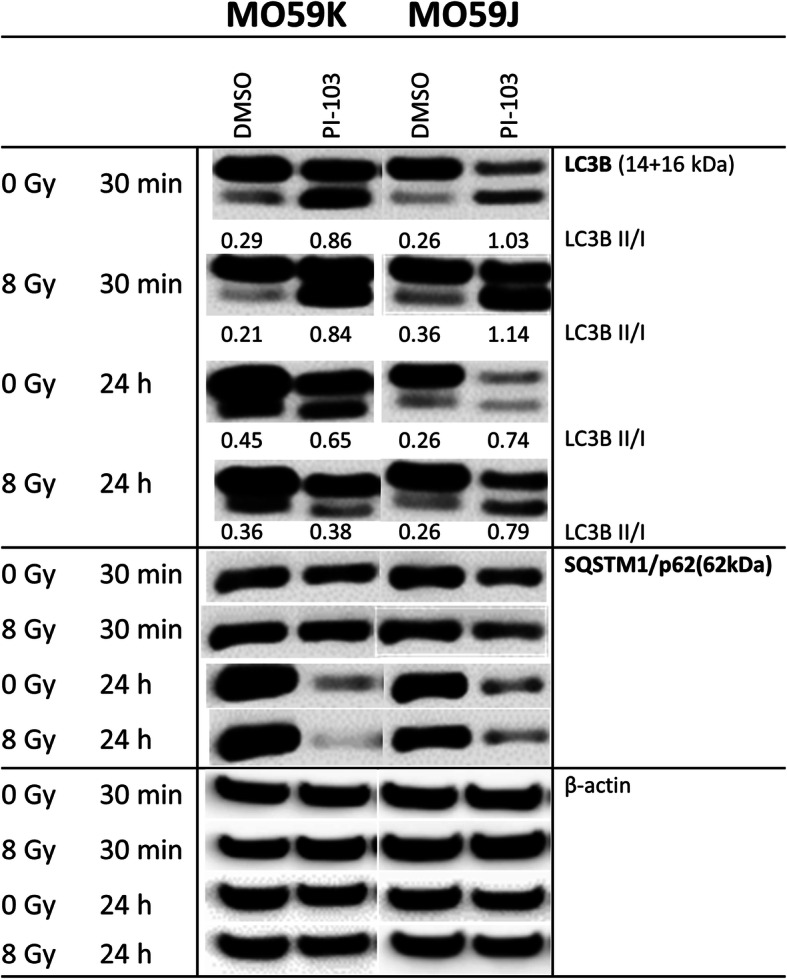
Western blot analysis of autophagy marker proteins LC3B und p62 in MO59K and MO59J cells treated with DMSO (control) or PI-103 for 3 h prior to IR with 8 Gy and detected 30 min and 24 h thereafter. The uncropped blots are shown in Fig. S12. β-actin was used as loading control. The experiments were repeated at least three times. For details, *see* legend to Fig. 2

Because the PI3K pathway is a major pathway regulating autophagy [40], we also studied the possible role of cytoprotective autophagy in the development of radiation resistance in PI-103-treated MO59J cells (Fig. 1). To this end, we detected the autophagosomal membrane-bound LC3B protein along with the expression of the p62/sequestome protein, a pleiotropic protein that is consumed during autophagy [41]. Interestingly, we found that PI-103 added for 3 h strongly induced autophagy, as evident from the increased levels of LC3B-II protein in both cell lines (Fig. 7). However, prolonged incubation with PI-103 increased autophagy only in MO59J cells. These findings agree well with the results of Fan et al. (2010) who found that dual inhibition of PI3K and mTOR promotes survival of glioma cells by inducing cytoprotective autophagy [42]. Furthermore, the enhanced autophagy in PI-103-treated (24 h) MO59J samples, indicated by LC3B-II expression, was also corroborated by the strong reduction of p62, another marker of autophagy (Fig. 7) in MO59J cells treated with PI-103. In contrast, the reduction of p62 in MO59K cells treated with PI-103 (24 h post-IR) was not correlated with the LC3B-II marker. The highest extent of autophagy assessed by p62 expression was observed in samples treated with PI-103 alone. To sum up, a 24-h treatment with PI-103 induced autophagy in MO59J cells as evidenced by both markers. In contrast, the respective samples of MO59K cells showed increased autophagy only in case of p62 marker. It is worth to be mentioned, however, that the measurement of p62 expression strictly as a marker of autophagic flux is still controversial and can be misinterpreted mainly because this protein is subject to complex regulation at both the transcriptional and post-translational levels [43]. Similarly, the increase of the LC3B-II marker can be interpreted as an inhibition of autophagic flux.

### Effects of PI-103 and irradiation on the cell-cycle progression

**Fig. 8:**
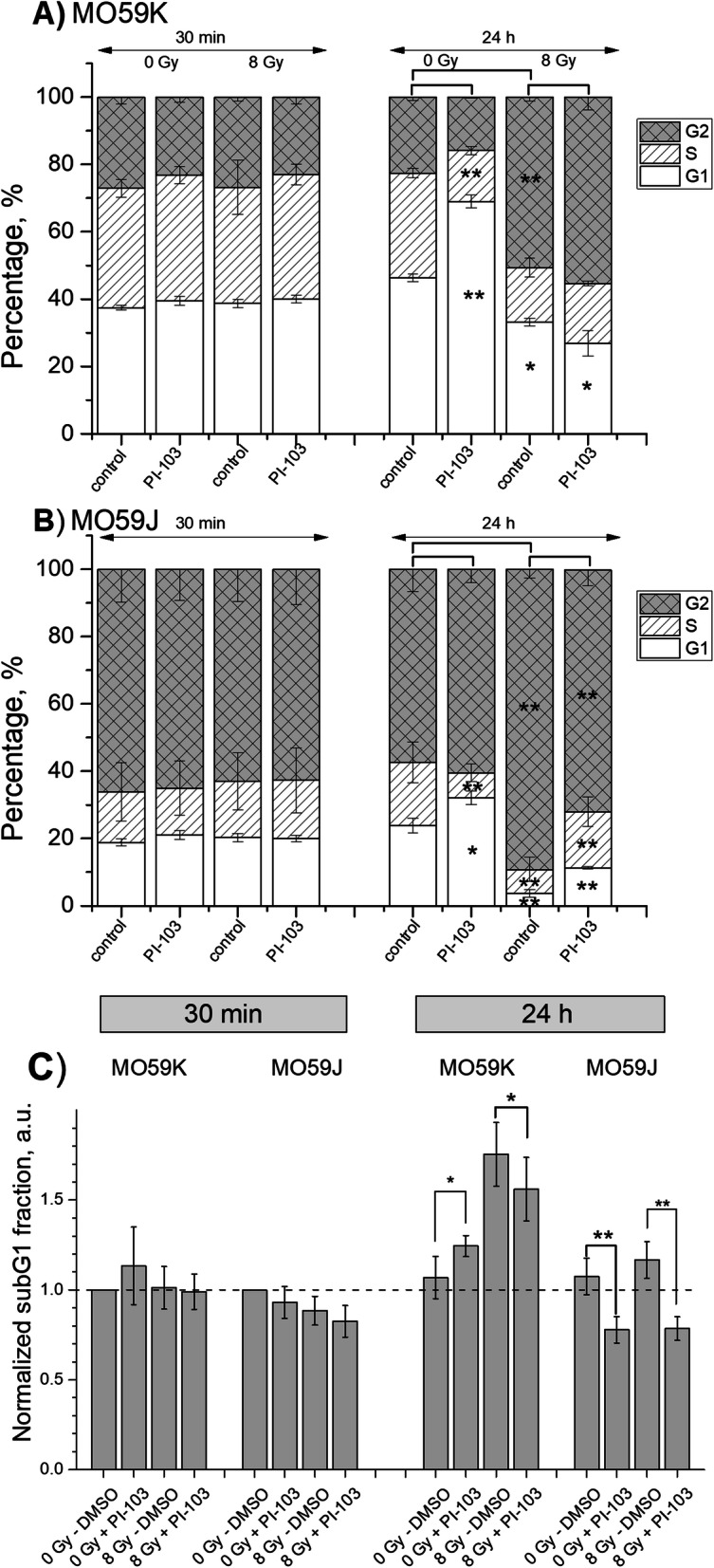
Cell cycle-phase distribution in MO59K (**a**) and MO59J (**b**) tumor cells treated for 3 h with PI-103 and irradiated with 8 Gy. Thirty minutes and 24 h after IR cells were fixed, permeabilized, stained with PI, and analyzed for DNA content by flow cytometry. Part **c** shows normalized fractions of cells with hypodiploid DNA content and cellular debris (subG1 fraction) in drug-treated and irradiated GBM cell lines measured flow-cytometrically. The percentage of subG1 fraction in non-irradiated control cell samples was set to unity. Data are presented as means (± SE) of at least three independent experiments

Besides apoptosis, the most prominent consequence of p53 activation is cell-cycle arrest [44]. We found that the long-term treatment with PI-103 caused a reduction of S phase and an accumulation of cells in G1 phase (70%) in non-irradiated MO59K cells, whereas irradiated and PI-103-treated MO59K cells showed a strong G2-arrest (45–55%) and a reduction of G1-fraction (Fig. 8a). Unlike MO59K cells, drug-free MO59J cells showed strong G2-arrest (~ 80–90%) 24 h after irradiation (Fig. 8b). Interestingly, addition of PI-103 significantly reduced G2-arrest in irradiated MO59J cells and increased the fractions of G1- and S-phase cells, which is indicative of the partial abolishment of cell-cycle arrest. From the cell cycle measurements we extracted the data on the subG1 fraction in the samples (Fig. 8c). As seen in Fig. 8C, prolonged incubation with PI-103 in non-irradiated and irradiated MO59J cells *reduced* the subG1 fraction in these samples. This finding corroborates the increased radiation survival of PI-103-treated MO59J (Fig. 1).

## Discussion

In this study, we tested the effects of targeting the PI3K pathway using the triple DNA-PK/PI3K/mTOR inhibitor PI-103 in combination with IR to induce radiosensitization in two GBM cell lines differing in DNA-PK function [29]. The SF2 values obtained here (Fig. 1 and Additional file 1: Table S1) revealed that MO59J cells were ~ 4 times more sensitive to IR as compared to MO59K cells, which is in qualitative agreement with the radiosensitivity data for these cell lines reported elsewhere [29]. Unexpectedly we found that the DNA-PK/PI3K/mTOR-inhibitor PI-103 affected radiosensitivity in a cell line-dependent manner, i.e. it acted as a radiosensitizer in MO59K cells, but it induced radiation resistance in MO59J cells (Fig. 1). The index of drug-IR combination for clonogenic MO59K cell viability was determined to be lower than 0.7 thus giving a synergism of radiation and PI-103 treatments (Additional file 1: Fig. S6). This drug-IR synergism in MO59K cells indicates a radiosensitizing effect of PI-103. The effect of PI-103 was found to be independent of extracellular matrix (Additional file 1: Fig. S7). In addition, we studied the expression of several marker proteins involved in cell adhesion, such as FAK, p-FAK (Ser910), ILK1, RhoA, cdc42 and Rac1/2/3 proteins (Additional file 1: Fig. S8). As seen in Fig. S8, the background expression levels of the analyzed proteins were very similar in both cell lines, and neither radiation nor PI-103 significantly affected the expression of the tested proteins.

**Fig. 9:**
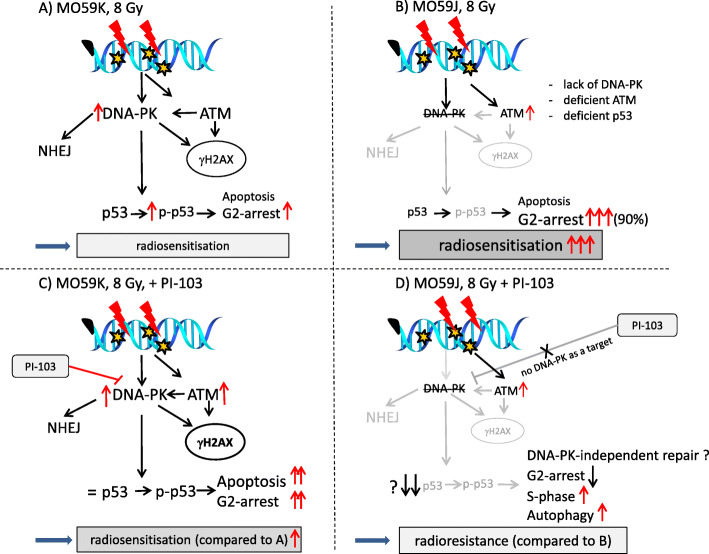
A simplified diagram of putative signaling pathways accountable for the differential responses of MO59K (**a**, **c**) and MO59J (**b**, **d**) cells to DNA-PK/PI3K/mTOR-inhibition and IR. Irradiation of MO59K cells in the presence of PI-103 showed increased radiation sensitivity (**c**) compared with the irradiated drug-free controls (**a**). Intrinsically radiosensitive MO59J cell line (**b**) is characterized by the absence of DNA-PK, and deficiencies in ATM and in NHEJ-repair. Both DNA-PK and ATM redundantly phosphorylate similar substrates, e.g. both are required for normal levels of p53 phosphorylation and p53-dependent apoptosis [37]. Accordingly, we found diminished expression of p53 in MO59J cells, which was further reduced in the presence of PI-103. Subsequently, the reduction of p53 might impede two most prominent outcomes of p53 function, i.e. cell-cycle arrest and apoptosis. Indeed, PI-103-treated and irradiated MO59J cells showed less apoptosis, reduced G2-arrest, increased S-phase fraction and cytoprotective authophagy compared with the irradiated drug-free MO59J cells (**b**). The findings might explain PI-103-induced radioresistance in MO59J cells (**d**) compared with the irradiated drug-free controls (**b**). (Note the size of the letters/symbols and the thickness of the lines, indicating expression levels). For details, *see* text

Figure 9 outlines our key findings. At the time of IR, both cell lines treated with PI-103 were strongly depleted of p-Akt. Surprisingly, long-term (24 h) incubation with PI-103 led to the *re-activation* of p-Akt in MO59K cells (Fig. 2, LHS). Activated Akt is widely recognized as the major mediator of cell survival, which inhibits apoptosis through several mechanisms [45], e.g. preserving mitochondrial integrity, phosphorylation and inactivation of pro-apoptotic BAD (Bcl-2-antagonist of cell death) and caspase 9 etc. [46]. BAD maintains Bcl-2 (B-cell lymphoma 2) and Bcl-xL function thereby inhibiting apoptosis mainly at the mitochondrial level by suppressing cytochrome *c* release [47]. However, despite re-activation of Akt and p-mTOR, the clonogenic survival data (Fig. 1) showed that PI-103 decreased radiation survival of MO59K cells. In contrast, radiation survival of PI-103-treated MO59J cells was unexpectedly *increased*, although in these samples the p-Akt protein was reactivated to a much lesser extent (Fig. 2, RHS) than that of MO59K cells. The same samples of MO59J cells showed strong up-regulation of p-Erk1/2 (Additional file 1: Fig. S6, LHS) which could explain, at least partly, the radioresistance of PI-103-treated MO59J cells.

A further factor of chemically or radiation-induced cell resistance or death is the induction and repair of DNA DSBs, probed in this study by counting of γH2AX and 53BP1 foci (Figs. 3a-d). γH2AX and 53BP1 foci counting revealed some differences in the foci induction rate between the two cell lines. Yet, the initial radiation-induced foci amounts were very similar, which is in line with the finding of Stiff et al. (2004) [30]. However, if the γH2AX foci counts (Fig. 3) were corrected for the cell line-specific chromosome content [16], MO59J cells would display a lower γH2AX foci-to-chromatin ratio than MO59K cells.

We found a strong difference in the kinetics of γH2AX and 53BP1 induction and disappearance between irradiated controls of both cell lines (Fig. 3c and d). Particularly, MO59J cells displayed a slower induction of γH2AX foci (Fig. 3d) as compared to MO59K cells (Fig. 3c), which is in accordance with the lack of NHEJ in MO59J cells and their high radiation sensitivity [16]. However, after treating MO59K cells with PI-103 the γH2AX and 53BP1 disappearance was delayed (Fig. 3c), which could explain the increased radiation toxicity as compared to control MO59K samples (Fig. 1a). Despite the strong induction of γH2AX and 53BP1 foci in irradiated and drug-treated MO59J cells (Fig. 3d), the residual number of foci was almost the same as that in drug-free irradiated MO59J cells and in MO59K cells treated with PI-103. Accordingly, Wang et al. (1997) found no marked difference in the unwinding/rewinding of DNA supercoils, radiation-induced changes in nucleoid halo size or the kinetics of nucleoid halo lysis between these two cell lines [48]. Likewise, an efficient rejoining of DNA DSBs has been observed in vitro in extracts of MO59J cells, despite the lack of DNA-PK activity in this strain [49].

Given that H2AX is phosphorylated to γH2AX, among other factors, by DNA-PK and ATM [30], and exactly these two proteins are either completely absent (DNA-PK) or reduced (ATM) in MO59J cells, it is conceivable that DNA damage assessment based on γH2AX detection underestimates the actual degree of DNA damage, at least in MO59J cells. This line of reasoning is supported by our preliminary tests, which revealed strong difference in chromatin compactness between the two tested cell lines (Additional file 1: Figs. S1-S4). Another reason may be a partial enhancement of the HR pathway in PI-103 treated MO59J cells as compared to control (Fig. 5). In addition, as suggested by Virsik-Köpp and co-workers (2003), the elimination of DNA-PK-dependent NHEJ can recruit a slow, error-prone repair process, which is DNA-PK independent [16].

Another possible reason for the opposite effects of PI-103 on the radiation survival of the two tested cell lines might be a difference in their proneness to PI-103-induced apoptosis. The DNA repair protein DNA-PKcs and the signal transducer ATM are both known to be activated by DNA DSBs [37]. Both DNA-PK and ATM redundantly phosphorylate similar substrates, e.g. both are required for normal levels of p53 phosphorylation and p53-dependent apoptosis [37]. Indeed, our experiments revealed a diminished expression level of p53 and lack of p-p53 in MO59J cells (Fig. 6). Accordingly, we found higher expression of the apoptosis marker cleaved PARP in MO59K cells treated with PI-103 (30 min post-IR) compared to respective samples of MO59J cells (Additional file 1: Fig. S7).

Although DNA-PK is an important DNA repair protein, it can also influence gene expression by phosphorylation of various transcription factors including Fos, Jun, myc, and p53, which are known to regulate either cell death or cell growth [50]. There are conflicting data on the role of DNA-PK in regulation of cell death. Thus, DNA-PK has been reported *to protect* cells from death via a caspase-independent [51] or p53-independent [52] pathway. On the other hand, DNA-PK can *promote* cell death by interacting with telomeres, forming a complex with p53, and phosphorylating MDM2 to render it unable to inhibit p53 transactivation [53].

Our findings clearly indicate a cell death-promoting role of DNA-PK. Thus, MO59K cells containing intact DNA-PK were more sensitive to combined PI-103 and IR treatment (Fig. 1) than to IR alone, whereas MO59J cells lacking DNA-PK were more resistant to the combined PI-103-IR treatment than to IR alone. The reasons for these divergent findings might involve a variety of factors.

As known, the most prominent outcomes of p53 activation are apoptosis and cell-cycle arrest [44]. We found that the expression of pro-apoptotic Bax was much higher in MO59K cells than in MO59J cells, especially after addition of PI-103 (Fig. 6). Given that Bax is related to p53, this finding is in line with the pro-apoptotic function of p53 and its different expression levels in both cell lines (Fig. 6). Moreover, our results are consistent with the p53-regulating function of DNA-PK [50]. It is well known that p53 is able to up-regulate Bax in variety of cell types including glioma [54]. Therefore, it is reasonable to assume that the decreased expression of the pro-apoptotic protein Bax in MO59J cells may be due to reduced level of p53, which is known to result from deficiency of DNA-PK [55]. Another consequence of the diminished expression of p53 and the absence of the activated form of p53 (p-p53) in MO59J cells is a strong alteration of the cell cycle, i.e. the majority (~ 80%) of irradiated drug-free MO59J cells were arrested in G2-phase. However, IR treatment in the presence of PI-103 resulted in a partial cell-cycle progression of MO59J cells, as evidenced by reduction of the G2 fraction and doubling of the S-phase fraction. This might be another reason for the increased radiation survival of MO59J cells treated with PI-103 as compared to the drug-free irradiated controls.

In accordance with our findings, the pro-cell death role of DNA-PK in human glioblastoma was demonstrated by Chakravarty et al. (1999) who showed that activation of DNA-PK by staurosporine, ceramide or UV radiation increases apoptosis in human neuroblastoma cells [56]. Likewise, Chen et al. (2005) found that MO59K cells were much more sensitive to staurosporine treatment than MO59J cells [50], which corroborates our results on the sensitivity of MO59J cells to combined PI-103-IR treatment.

An additional reason for the opposing effect of PI-103 on the radiation sensitivity of the tested GBM cell lines could be protective autophagy. As known, PI3K signaling is a major pathway regulating autophagy [40]. Indeed, we found an increased autophagy indicated by LC3B-II and p62 markers in irradiated PI-103-treated MO59J cells (Fig. 7).

## Conclusions

To sum up, our data demonstrate an enhanced radiosensitivity in MO59K tumor cells pretreated with the triple DNA-PK/PI3K/mTOR inhibitor PI-103 added shortly before IR. In contrast, the same treatment caused radioresistance in MO59J cells, which lack one of the target proteins for PI-103, i.e. DNA-PK. However, the lack of the NHEJ pathway in DNA-PK deficient MO59J cells might have been partially compensated by a slow, DNA-PK independent and yet unexplored DNA repair pathway. Another explanation for the radioresistance of PI-103-treated MO59J cells might be their DNA-PK deficiency, leading to diminished expression and activation of the DNA-PK substrate p53. PI-103 treatment further reduced p53 expression in MO59J cells, resulting in a resistance to apoptosis. In contrast, intact DNA-PK in MO59K cells treated with PI-103 *activated* p53 and related pro-apoptotic proteins. Moreover, the massive G2 arrest in irradiated MO59J cells was partially abolished by PI-103, thus allowing cell-cycle progression of a significant portion of cells. Unlike in MO59J cells, the G2 fraction in irradiated and PI-103-treated MO59K cells was increased. Furthermore, DNA damage assessed by γH2AX expression in Western blot showed a strong difference between the two cell lines. Thus, irradiation in the presence of PI-103 caused a much stronger induction of γH2AX in MO59K cells than in MO59J cells. In conclusion, to further elucidate the mechanisms of radiosensitization in DNA-PK- and ATM-deficient tumor cells, studies on an extended panel of cell lines including genetically manipulated cell lines with PTEN- or p53-knockout, regarding the cell type-specific activation of p53, cell-cycle arrest, apoptosis and DNA repair will be the subject of future research in our laboratory.

## Supplementary Information

**Additional file 1: Supplemental Materials.** The primary and secondary antibodies used in this paper. **Supplemental Methods.**: Comet assay and staining for HDAC5 and DAPI. In order to characterize the tested cell lines, preliminary experiments were carried out via Comet assay. The Comet assay was performed under alkaline conditions following a protocol reported elsewhere [19]. Just before irradiation, cells were embedded in a thin layer of agarose spread on glass microscope slides. The slides were placed on ice, subjected to irradiation and transferred immediately either into ice-cold lysis buffer or in CGM for the indicated times. DNA fragmentation was quantified from the “Tail Moment” (TM, given in arbitrary units, a.u.) defined as the product of the percentage of DNA in the comet tail and the tail length. Supplementary Fig. S1 shows the induction and repair of DNA damage measured by the Comet assay in the cells of both cell lines immediately and up to 40 min after irradiation with 5 Gy. As seen in Supplementary Fig. S1, the initial TM was higher in MO59J cells, however, the residual DNA damage was similar in both cell lines, as were the kinetics of DNA damage disappearance. Supplementary Fig. S2 shows the distribution of TM in both cell lines measured immediately after IR. As seen in Supplementary Fig. S2, the histogram of TM values in intrinsically radiosensitive MO59J line was shifted towards higher DNA damage. It is worth mentioning that DNA damage measured by the alkaline Comet assay includes single-strand breaks and alkali-labile sites and base exchanges. Another preliminary test to characterize intrinsically radioresistant MO59K and radiosensitive MO59J cell lines was staining for HDAC5 protein and subsequent HDAC5 foci counting (Supplementary Fig. S3). To this end, cells were cultured on microscope glass slides for at least 24 h and stained with anti-HDAC5 (1:200) primary antibodies essentially as described elsewhere [57] with the exception of primary antibodies. For each experiment or cell line, at least 100 nuclei were examined and HDAC5 foci were scored by eye at a magnification of 1000x. Probes were then quantified by counting the number of foci per nucleus. As seen in Supplementary Fig. S3, HDAC5 foci were much more expressed in radioresistant MO59K cell line. Simultaneously slides were counterstained with DAPI (Supplementary Fig. S4). **Fig. S1.** DNA damage (Tail Moment, TM) induction and repair measured by the comet assay in human GBM cell lines irradiated with 5 Gy of X-rays in vitro. Immediately after irradiation, the samples were placed at 37 °C in a 5% CO_2_ incubator. At the indicated time intervals after X-ray exposure, the cells were lysed and subjected to the alkaline comet assay. Up to 75 cells were analyzed for each slide. Each point (bar) represents the mean value (± SE) of TM for the respective time point. The curves are best least-square fits of exponential decay function to the datapoints. **Fig. S2.** DNA damage (Tail Moment) induction measured by the comet assay in human GBM cell lines MO59K and MO59J irradiated with 5 Gy of X-rays in vitro. The cells taken at the indicated time (2 min) after X-ray exposure were lysed and subjected to the alkaline Comet assay. Up to 75 cells were analyzed for each slide. The curves are best least-square fits of normal distribution to the datapoints. **Fig. S3.** Representative histograms depicting the HDAC5 focus formation in control non-irradiated MO59K and MO59J cells. Cells were analyzed for HDAC5 focus formation 24 h after seeding. About 100 nuclei were counted per each cell line and experiment (*n* = 3). **Fig. S4.** Representative histograms depicting the mean DAPI fluorescence intensity per nucleus in control non-irradiated MO59K and MO59J cells. Cells were cultured on the slides and 24 h after seeding stained with DAPI. Fluorescence intensity was quantitated with the ImageJ program in about 100 nuclei counted per each cell line and experiment (*n* = 3). **Fig. S5.** Cellular viability measured by an ATP test. Changes of intracellular ATP in 2 tumor cell lines exposed to serial dilutions of PI-103 for 24 h. ATP content was measured by standard luciferase bioluminescence assay. Quadruplicate data derived from at least three independent experiments were averaged, normalized against non-treated controls (DMSO) and analyzed using the standard four-parameter logistic model to generate dose-response curves. Error bars indicate SD values. **Fig. S6.** Effects of radiation, PI-103 or combined PI-103-radiation treatments on colony-forming ability of MO59K cells. One thousand MO59K cells were plated on Petri dishes for 16 h, treated with radiation (**A**: 2, 3, and 5 Gy), or with PI-103 (**B**: 0.5 μM, 1 μM, and 2 μM) or a combination of radiation (**C**: 3 Gy) and PI-103 (0.5 μM, 1 μM and 2 μM)) added 3 h before irradiation. Twenty four hours post-irradiation the inhibitor was washed out and the cultures were incubated in fresh CGM for the next 12 days, fixed and stained with crystal violet. The numbers of colonies were counted and averaged from three independent experiments. Combination index was determined according Malyarenko et al. (2020) and Chou (2010) and found to be at tested concentrations of PI-103 and radiation dose of 3 Gy < 0.7. Therefore, the combined effect of radiation and PI-103 was synergistic [23, 24]. **Fig. S7.** Comparison of substrate-dependent clonogenic cell survival. MO59K (**A**) and MO59J (**B)** cells were plated either on polystyrene (control, uncoated), fibronectin (FN) or BSA coated Petri dishes, exposed to DMSO (control) or PI-103 and/or irradiated, and the clonogenic survival was determined as shown in Fig. 1d, e. **Fig. S8.** Representative Western blot analysis of expression levels of several adhesion-associated proteins in MO59K and MO59J tumor cells treated with DMSO (control) or PI-103 for 3 h prior to IR with 8 Gy and detected 30 min and 24 h thereafter. β-actin was used as loading control. The experiment was repeated at least three times. For details, *see* legend to Fig. 2. **Fig. S9.** Representative Western blot analysis of expression levels of several marker proteins of MAPK-pathway in MO59K and MO59J tumor cells treated with DMSO (control) or PI-103 for 3 h prior to IR with 8 Gy and detected 30 min and 24 h thereafter. The experiment was repeated at least three times. For details, *see* legend to Fig. 2. **Fig. S10.** Western blot analysis of PARP and cleaved PARP proteins in MO59K and MO59J tumor cells treated with DMSO (control) or PI-103 for 3 h prior to IR with 8 Gy and detected 30 min and 24 h thereafter. β-actin was used as loading control. The experiment was repeated at least three times. For details, *see* legend to Fig. 2. **Fig. S11.** Normalized expression of marker proteins (p-Akt, p-4E-BP1, p-S6, LC3B II/I and SQSTM1/p62) in MO59K and MO59J cells treated with DMSO (control) or PI-103 for 3 h prior to IR with 8 Gy and detected 30 min and 24 h thereafter. The experiments were repeated at least three times, mean values ± SE are shown. Differences between PI-103 and/or IR treatment were statistically analyzed using Student’s *t*-test. The mean value of protein content in control non-irradiated sample was set to 1. For details, *see* legend to Fig. 2. **Table S1.** Cloning efficiencies and radiosensitivity parameters^a^ of in vitro irradiated tumor cell lines untreated and pretreated (3 h) prior to IR with the PI-103 and re-plated 24 h post-IR. ^a^Mean (± SE) from at least three independent experiments; ^b^SF2 is the colony-forming ability at 2 Gy; ^c^D_10_ is the radiation dose required to reduce colony-forming ability by 10%; ^d^The growth inhibition factor IF_10_ was calculated as (D_10_ control)/(D_10_ + inh.); ^*^means statistical significance of the differences at *P* < 0.05.

**Additional file 2: Supplementary Fig. S12.** Uncropped representative Western blots for Figs. 2-6, and Supplementary Figs. S8-S10. The figures show original blots, in some cases the same membrane was used to simultaneously detect different proteins (after cutting) which strongly deviated in molecular weight. All original blots for β-actin used as loading control for the respective runs are also included.

## Data Availability

All data generated and/or analyzed during this study are included in this published article and its Additional files.

## Abbreviations

BSA: Bovine serum albumin
CGM: Complete growth medium
CI: Combination index
DAPI: 4′,6′-diamidino-2-phenylindole
D_10_: Radiation dose required to reduce clonogenic survival by 10%
DSB: Double-strand breaks
Erk: Extracellular signal-regulated kinase
ECM: Extracellular matrix
FN: Fibronectin
Fpn: Foci per nucleus
GBM: Glioblastoma
HR: Homologous recombination
HDAC5: Histone deacetylase 5
IR: Ionizing radiation
LHS: Left-hand side
LQ: Linear-quadratic
MAPK: Mitogen-activated protein kinase (MAPK) kinase (MEK)
MEK: Mitogen-activated protein kinase (MAPK) kinase
NHEJ: Non-homologous end-joining
PE: Plating efficiency
PI: Propidium iodide
PI3K: Phosphoinositid-3-kinase
RAF: Rat fibrosarcoma protein
RAS: Rat sarcoma protein
RHS: Right-hand side
RT: Radiation therapy
SF2: Surviving fraction at 2 Gy

## References

[1] Webb S (2000). Advances in three-dimensional conformal radiation therapy physics with intensity modulation. Lancet Oncol.

[2] Boon IS, Au Yong TPT, Boon CS (2018). Assessing the role of artificial intelligence (AI) in clinical oncology: utility of machine learning in radiotherapy target volume delineation. Medicines (Basel).

[3] Stupp R, Mason WP, van den Bent MJ, Weller M, Fisher B, Taphoorn MJ, Belanger K, Brandes AA, Marosi C, Bogdahn U, Curschmann J, Janzer RC, Ludwin SK (2005). Radiotherapy plus concomitant and adjuvant temozolomide for glioblastoma. N Engl J Med.

[4] Vokes EE, Haraf DJ, Brockstein BE, Weichselbaum RR (1999). Paclitaxel, 5-fluorouracil, hydroxyurea, and concomitant radiotherapy for poor-prognosis head and neck cancer. Semin Radiat Oncol.

[5] Milas L, Mason KA, Liao Z, Ang KK (2003). Chemotherapy: emerging treatment improvement strategies. Head Neck.

[6] Weichselbaum RR (1995). Growth factors alter the therapeutic ratio in radiotherapy. Cancer J Sci Am.

[7] Chmura SJ, Gupta N, Advani SJ, Kufe DW, Weichselbaum RR (2001). Prospects for viral-based strategies enhancing the anti-tumor effects of ionizing radiation. Semin Radiat Oncol.

[8] Dasika GK, Lin SC, Zhao S, Sung P, Tomkinson A, Lee EY (1999). DNA damage-induced cell cycle checkpoints and DNA strand break repair in development and tumorigenesis. Oncogene..

[9] Green CM, Almouzni G (2002). When repair meets chromatin. EMBO J.

[10] Smerdon MJ, Conconi A (1999). Modulation of DNA damage and DNA repair in chromatin. Prog Nucleic Acids Res Mol Biol.

[11] Rogakou EP, Pilch DR, Orr AH, Ivanova VS, Bonner WM (1998). DNA double-stranded breaks induce histone H2AX phosphorylation on serine 139. J Biol Chem.

[12] Olive PL, Banáth JP (2004). Phosphorylation of histone H2AX as a measure of radiosensitivity. Int J Radiat Oncol Biol Phys.

[13] Huyen Y, Zgheib O, Ditullio RA, Gorgoulis VG, Zacharatos P, Petty TJ, Emily Sheston A, Mellert HS, Stavridi ES, Halazonetis TD (2004). Methylated lysine 79 of histone H3 targets 53BP1 to DNA double-strand breaks. Nature.

[14] Dienstmann R, Rodon J, Serra V, Tabernero J (2014). Picking the point of inhibition: a comparative review of PI3K/AKT/mTOR pathway inhibitors. Mol Cancer Ther.

[15] Engelman JA (2009). Targeting PI3K signalling in cancer: opportunities, challenges and limitations. Nat Rev Cancer.

[16] Virsik-Köpp P, Rave-Fränk M, Hofman-Hüther H, Schmidberger H (2003). Role of DNA-PK in the process of aberration formation as studied in irradiated human glioblastoma cell lines M059K and M059J. Int J Radiat Biol.

[17] Michaud K, Solomon DA, Oermann E, Kim J-S, Zhong W-Z, Prados MD, Ozawa T, James CD, Waldman T (2010). Pharmacologic inhibition of cyclin-dependent kinases 4 and 6 arrests the growth of glioblastoma multiforme intracranial xenografts. Cancer Res.

[18] Cann KL, Dellaire G (2011). Heterochromatin and the DNA damage response: the need to relax. Biochem Cell Biol.

[19] Djuzenova CS, Rothfuss A, Oppitz A, Speit SD, Hoehn H, Flentje M (2001). Response to X-irradiation of Fanconi anemia homozygous and heterozygous cells assessed by the single-cell gel electrophoresis (comet) assay. Lab Investig.

[20] Cress WD, Seto E (2000). Histone deacetylases, transcriptional control, and cancer. J Cell Physiol.

[21] Mascetti G, Carrara S, Vergani L. Relationship between chromatin compactness and dye uptake for in situ chromatin stained with DAPI. Cytometry. 2001;44(2):113–9. 10.1002/1097-0320(20010601)44:2<113::aid-cyto1089>3.0.co;2-a. 10.1002/1097-0320(20010601)44:2<113::aid-cyto1089>3.0.co;2-a 11378861

[22] Djuzenova CS, Fiedler V, Katzer A, Michel K, Deckert S, Zimmermann H (2016). Dual PI3K- and mTOR-inhibitor PI-103 can either enhance or reduce the radiosensitizing effect of the Hsp90 inhibitor NVP-AUY922 in tumor cells: the role of drug-irradiation schedule. Oncotarget.

[23] Chou T-C (2010). Drug combination studies and their synergy quantification using the Chou-Talalay method. Cancer Res.

[24] Malyarenko OS, Imbs TI, Ermakova SP (2020). In vitro anticancer and radiosensitizing activities of phlorethols from the brown alga Costaria costata. Molecules..

[25] Cordes N, Hansmeier B, Beinke C, Meineke V, van Beuningen D (2003). Irradiation differentially affects substratum-dependent survival, adhesion, and invasion of glioblastoma cell lines. Br J Cancer.

[26] Sisario D, Memmel S, Doose S, Neubauer S, Zimmermann H, Flentje M, Djuzenova CS, Sauer M, Sukhorukov VL (2018). Nanostructure of DNA repair foci revealed by superresolution microscopy. FASEB J.

[27] Djuzenova CS, Marcus Zimmermann M, Katzer A, Fiedler V, Distel LV, Gasser M (2015). A prospective study on histone γ-H2AX and 53BP1 foci expression in rectal carcinoma patients: correlation with radiation therapy-induced outcome. BMC Cancer.

[28] Memmel S, Sisario D, Zimmermann H, Sauer M, Sukhorukov VL, Djuzenova CS, Flentje M (2020). FocAn: automated 3D analysis of DNA repair foci in image stacks acquired by confocal fluorescence microscopy. BMC Bioinformatics.

[29] Allalunis-Turner MJ, Zia PK, Baron GM, Mirzayans R, Day RS (1995). Radiation-induced DNA damage and repair in cells of a radiosensitive human malignant glioma cell line. Radiat Res.

[30] Stiff T, O’Driscoll M, Rief N, Iwabuchi K, Löbrich M, Jeggo PA (2004). ATM and DNA-PK function redundantly to phosphorylate H2AX after exposure to ionizing radiation. Cancer Res.

[31] Fingar DC, Richardson CJ, Tee AR, Cheatham L, Tsou C, Blenis J (2004). mTOR controls cell cycle progression through its cell growth effectors S6K1 and 4E-BP1/eukaryotic translation initiation factor 4E. Mol Cell Biol.

[32] Foster DA, Yellen P, Xu L, Saqcena M (2010). Regulation of G1 cell cycle progression: distinguishing the restriction point from a nutrient-sensing cell growth checkpoint(s). Genes Cancer.

[33] McCubrey JA, Steelman LS, Chappell WH, Abrams SL, Wong EWT, Chang F (2007). Roles of the Raf/MEK/ERK pathway in cell growth, malignant transformation and drug resistance. Biochim Biophys Acta.

[34] Steelman LS, Chappell WH, Abrams SL, Kempf RC, Long J, Laidler P (2011). Roles of the Raf/MEK/ERK and PI3K/PTEN/Akt/mTOR pathways in controlling growth and sensitivity to therapy-implications for cancer and aging. Aging (Albany NY).

[35] Samatar AA, Poulikakos PI (2014). Targeting RAS-ERK signalling in cancer: promises and challenges. Nat Rev Drug Discov.

[36] Sedelnikova OA, Bonner WM (2006). Gamma H2AX in cancer cells: a potential biomarker for cancer diagnostics, prediction and recurrence. Cell Cycle.

[37] Callén E, Jankovic M, Wong N, Zha S, Chen HT, Difilippantonio S, Di Virgilio M, Heidkamp G, Alt FW, Nussenzweig A, Nussenzweig M (2009). Essential role for DNA-PKcs in DNA double-strand break repair and apoptosis in ATM-deficient lymphocytes. Mol Cell.

[38] De Vos M, Schreiber V, Dantzer F (2012). The diverse roles and clinical relevance of PARPs in DNA damage repair: current state of the art. Biochem Pharmacol.

[39] Wang S, Guo M, Ouyang H, Li X, Cordon-Cardo C, Kurimasa A, Chen DJ, Fuks Z, Ling CC, Li GC (2000). The catalytic subunit of DNA-dependent protein kinase selectively regulates p53-dependent apoptosis but not cell-cycle arrest. Proc Natl Acad Sci U S A.

[40] Vadlakonda L, Pasupuleti M, Pallu R (2013). Role of PI3K-AKT-mTOR and Wnt signaling pathways in transition of G1-S phase of cell cycle in cancer cells. Front Oncol.

[41] Karagounis IV, Kalamida D, Mitrakas A, Pouliliou S, Liousia MV, Giatromanolaki A, Koukourakis MI (2016). Repression of the autophagic response sensitises lung cancer cells to radiation and chemotherapy. Br J Cancer.

[42] Fan Q-W, Cheng C, Hackett C, Feldman M, Houseman BT, Nicolaides T (2010). Akt and autophagy cooperate to promote survival of drug-resistant glioma. Sci Signal.

[43] Puissant A, Fenouille N, Auberger P (2012). When autophagy meets cancer through p62/SQSTM1. Am J Cancer Res.

[44] Chen J (2016). The cell-cycle arrest and apoptotic functions of p53 in tumor initiation and progression. Cold Spring Harb Perspect Med.

[45] Marte BM, Downward J (1997). PKB/Akt: connecting phosphoinositide 3-kinase to cell survival and beyond. Trends Biochem Sci.

[46] Elkholi R, Renault TT, Serasinghe MN, Chipuk JE (2014). Putting the pieces together: how is the mitochondrial pathway of apoptosis regulated in cancer and chemotherapy?. Cancer Metab.

[47] Zhou H, Li XM, Meinkoth J, Pittman RN (2000). Akt regulates cell survival and apoptosis at a postmitochondrial level. J Cell Biol.

[48] Wang J, Hu L, Allalunis-Turner MJ, Day RS, Deen DF (1997). Radiation-induced damage in two human glioma cell lines as measured by the nucleoid assay. Anticancer Res.

[49] Cheong N, Perrault AR, Wang H, Wachsberger P, Mammen P, Jackson I, Iliakis G (1999). DNA-PK-independent rejoining of DNA double-strand breaks in humna cells extracts in vitro. Int J Radiat Biol.

[50] Chen GG, Sin FL, Leung BC, Ng HK, Poon WS (2005). Glioblastoma cells deficient in DNA-dependent protein kinase are resistant to cell death. J Cell Physiol.

[51] Chechlacz M, Vemuri MC, Naegele JR (2001). Role of DNA-dependent protein kinase in neuronal survival. J Neurochem.

[52] Gurley KE, Kemp CJ (1996). p53 induction, cell cycle checkpoints, and apoptosis in DNAPK-deficient scid mice. Carcinogenesis..

[53] Mayo LD, Turchi JJ, Berberich SJ (1997). Mdm-2 phosphorylation by DNA-dependent protein kinase prevents interaction with p53. Cancer Res.

[54] Pohl U, Wagenknecht B, Naumann U, Weller M (1999). p53 enhances BAK and CD95 expression in human malignant glioma cells but does not enhance CD95L-induced apoptosis. Cell Physiol Biochem.

[55] Achanta G, Pelicano H, Feng L, Plunkett W, Huang P (2001). Interaction of p53 and DNA-PK in response to nucleoside analogues: potential role as a sensor complex for DNA damage. Cancer Res.

[56] Chakravarthy BR, Walker T, Rasquinha I, Hill IE, MacManus JP (1999). Activation of DNA-dependent protein kinase may play a role in apoptosis of human neuroblastoma cells. J Neurochem.

[57] Djuzenova CS, Mühl B, Schakowski R, Oppitz U, Flentje M (2004). Normal expression of DNA repair proteins, hMre11, Rad50 and Rad51 but protracted formation of Rad50 fontaining foci in X-irradiated skin fibroblasts from radiosensitive cancer patients. Br J Cancer.

