# Supplementary material for: Opposite effects of the triple target (DNA-PK/PI3K/mTOR) inhibitor PI-103 on the radiation sensitivity of glioblastoma cell lines proficient and deficient in DNA-PKcs
Source: BMC Cancer. 2021 Nov 11;21:1201. doi: 10.1186/s12885-021-08930-1 (PMC8582108; doi:10.1186/s12885-021-08930-1)
Supplement: Supplementary file 1 — Additional file 1: Supplemental Materials. The primary and secondary antibodies used in this paper. Supplemental Methods.: Comet assay and staining for HDAC5 and DAPI. In order to characterize the tested cell lines, preliminary experiments were carried out via Comet assay. The Comet assay was performed under alkaline conditions following a protocol reported elsewhere [19]. Just before irradiation, cells were embedded in a thin layer of agarose spread on glass microscope slides. The slides were placed on ice, subjected to irradiation and transferred immediately either into ice-cold lysis buffer or in CGM for the indicated times. DNA fragmentation was quantified from the “Tail Moment” (TM, given in arbitrary units, a.u.) defined as the product of the percentage of DNA in the comet tail and the tail length. Supplementary Fig. S1 shows the induction and repair of DNA damage measured by the Comet assay in the cells of both cell lines immediately and up to 40 min after irradiation with 5 Gy. As seen in Supplementary Fig. S1, the initial TM was higher in MO59J cells, however, the residual DNA damage was similar in both cell lines, as were the kinetics of DNA damage disappearance. Supplementary Fig. S2 shows the distribution of TM in both cell lines measured immediately after IR. As seen in Supplementary Fig. S2, the histogram of TM values in intrinsically radiosensitive MO59J line was shifted towards higher DNA damage. It is worth mentioning that DNA damage measured by the alkaline Comet assay includes single-strand breaks and alkali-labile sites and base exchanges. Another preliminary test to characterize intrinsically radioresistant MO59K and radiosensitive MO59J cell lines was staining for HDAC5 protein and subsequent HDAC5 foci counting (Supplementary Fig. S3). To this end, cells were cultured on microscope glass slides for at least 24 h and stained with anti-HDAC5 (1:200) primary antibodies essentially as described elsewhere [57] with the exception of primary antibodi [file 12885_2021_8930_MOESM1_ESM.docx]

# Supplementary Materials

The primary antibodies used for Western blot were: rabbit monoclonal anti-PI3K p110α (C73F8), mouse monoclonal anti-phospho-Akt (Ser473) (D9E), rabbit monoclonal anti-phospho-mTOR (Ser2448) (D9C2), rabbit polyclonal anti-phospho-S6 ribosomal protein (Ser240/244), mouse monoclonal anti-phospho-4E-BP1 (Thr37/46) (236B4), rabbit monoclonal anti-phospho-MEK1/2 (Ser217/221) (41G9), rabbit monoclonal anti-HDAC5 (D1J7V), rabbit monoclonal anti-phospho-p44/42 MAPK (ERK1/2) (Thr202/Tyr204) (D13.14.4E), rabbit monoclonal anti-Rad51 (D4B10), rabbit polyclonal anti-PARP, mouse monoclonal anti caspase-3 (3G2 mouse monoclonal anti-phospho-p53 (Ser15)(16G8), rabbit monoclonal anti-Bax (D2E11), rabbit monoclonal anti-ATM (D2E2), Homologous Recombination (HR) DNA repair antibody sampler kit (99891), rabbit polyclonal anti-LC3B, rabbit monoclonal anti-SQSTM1/p62 (D5E2), rabbit monoclonal DNA-PKcs (E6U3A) rabbit polyclonal anti-Ku80, rabbit polyclonal anti-FAK, rabbit monoclonal anti-ILK1 (4G9), rabbit monoclonal anti-RhoA (67B9), rabbit polyclonal anti-cdc42  (all from Cell Signaling, Danvers, MA), mouse monoclonal anti-Rad50 (13B3/2C6), mouse monoclonal anti-p53 (DO-1), rabbit polyclonal anti-Raf-1 (C12) (Santa Cruz, Dallas, TX), mouse monoclonal anti-Ku70 (p70)Ab-4 (N3H10) rabbit polyclonal anti-FAK[pS^910^] (ThermoFisher Scientific, Wiesbaden, Germany), rabbit polyclonal anti-53BP1 (Novus Biologicals, Cambridge, UK), mouse monoclonal anti-β-actin (Sigma, Deisenhofen, Germany), mouse monoclonal anti-phospho-histone H2AX (Ser139) (Millipore, Schwalbach, Germany). Secondary species-specific antibodies for western blot were labelled with horseradish-peroxidase (DAKO, Hamburg, Germany). Secondary antibodies used for microscopy were: Alexa Fluor 532-conjugated goat anti-mouse IgG (ThermoFisher Scientific, Wiesbaden, Germany).

## Supplementary Methods

## Comet assay and staining for HDAC5 and DAPI

In order to characterize the tested cell lines preliminary experiments were carried out using Comet assay. The Comet assay was performed under alkaline conditions following the protocol reported elsewhere [17]. Shortly before irradiation, cells were imbedded in a thin layer of agarose spread on glass microscope slides. The slides were placed on ice, subjected to irradiation and transferred immediately either into ice-cold lysis buffer or in CGM for the indicated times. DNA fragmentation was quantified from the "Tail Moment" (TM, given in arbitrary units, a.u.) defined as the product of the percentage of DNA in the comet tail and the tail length. Supplementary Fig. S1 shows the induction and repair of DNA damage measured by the Comet assay in the cells of both cell lines immediately and up to 40 min after irradiation with 5 Gy. As seen in Supplementary Fig. S1, the initial TM was higher in the MO59J cells, however, the residual DNA damage was almost the same in both cell lines as well as the kinetics of its disappearance. Supplementary Fig. S2 shows the distribution of TM in both cell lines measured immediately after IR. As seen in Fig. S2, the histogram of TM values in intrinsically radiosensitive MO59J line were shifted towards the higher DNA damage. Notably, DNA damage measured by the alkaline Comet assay includes single-strand breaks and alkali-labile sites and base exchanges.

Another preliminary test to characterize intrinsically radioresistant MO59K and radiosensitive MO59J cells included staining for HDAC5 proteins and subsequent HDAC5 foci counting (Supplementary Fig. S3). To this end, cells were cultured on microscope glass slides for at least 24 h and stained with anti-HDAC5 (1:200) primary antibodies essentially as described elsewhere [Djuzenova et al., 2004] with the exception of primary antibodies. For each experiment or cell line, at least 100 nuclei were examined and HDAC5 foci were scored by eye at a magnification of 1000x. Samples were then quantified by counting the number of foci per nucleus. As seen in Supplementary Fig. S3, the HDAC5 foci were much more expressed in radioresistant MO59K cell line. Simultaneously slides were counterstained with DAPI (Supplementary Fig. S4).

|  |
| --- |
| **Figure S1.** DNA damage (Tail Moment, TM) induction and repair measured by the comet assay in human GBM cell lines irradiated with 5 Gy of X-rays *in vitro*. Immediately after irradiation the samples were placed at 37°C in a 5% CO_2_ incubator. The cells taken at the indicated time intervals after X-ray exposure were lysed and subjected to the alkaline comet assay. Up to 75 cells were analyzed for each slide. Each point (bar) represents the mean value (± SE) of TM for the respective time point. The curves are best least-square fits of an exponential decay function to the data points. |

|  |
| --- |
| **Figure S2.** DNA damage (Tail Moment) induction measured by the comet assay in the human GBM cell lines MO59K and MO59J irradiated with 5 Gy of X-rays *in vitro*. The cells taken at the indicated time (2 min) after X-ray exposure were lysed and subjected to the alkaline comet assay. Up to 75 cells were analyzed for each slide. The curves are best least-square fits of normal distribution to the data points. |

|  |
| --- |
| **Figure S3.** Representative histograms depicting the HDAC5 focus formation in control non-irradiated MO59K and MO59J cells. Cells were analyzed for HDAC5 focus formation 24 h after seeding. About 100 nuclei were counted per each cell line and experiment (n=3). |

|  |
| --- |
| **Figure S4.** Representative histograms depicting the mean DAPI fluorescence intensity per nucleus in control non-irradiated MO59K and MO59J cells. Cells were cultured on the slides and stained with DAPI 24 h after seeding. Fluorescence intensity was quantitated with the software ImageJ in about 100 nuclei counted per cell line and experiment (n=3). |

|  |
| --- |
| **Figure S5.** Cellular viability measured by an ATP test. Changes of intracellular ATP content in the human GBM cell lines MO59K and MO59J exposed to serial dilutions of PI-103 for 24 hours were measured against DMSO-treated controls. ATP content was measured by standard luciferase bioluminescence assay. Quadruplicate data derived from at least three independent experiments were averaged, normalized against non-treated controls (DMSO) and analyzed using the standard four-parameter logistic model to generate dose-response curves. Error bars indicate SD values. |

| 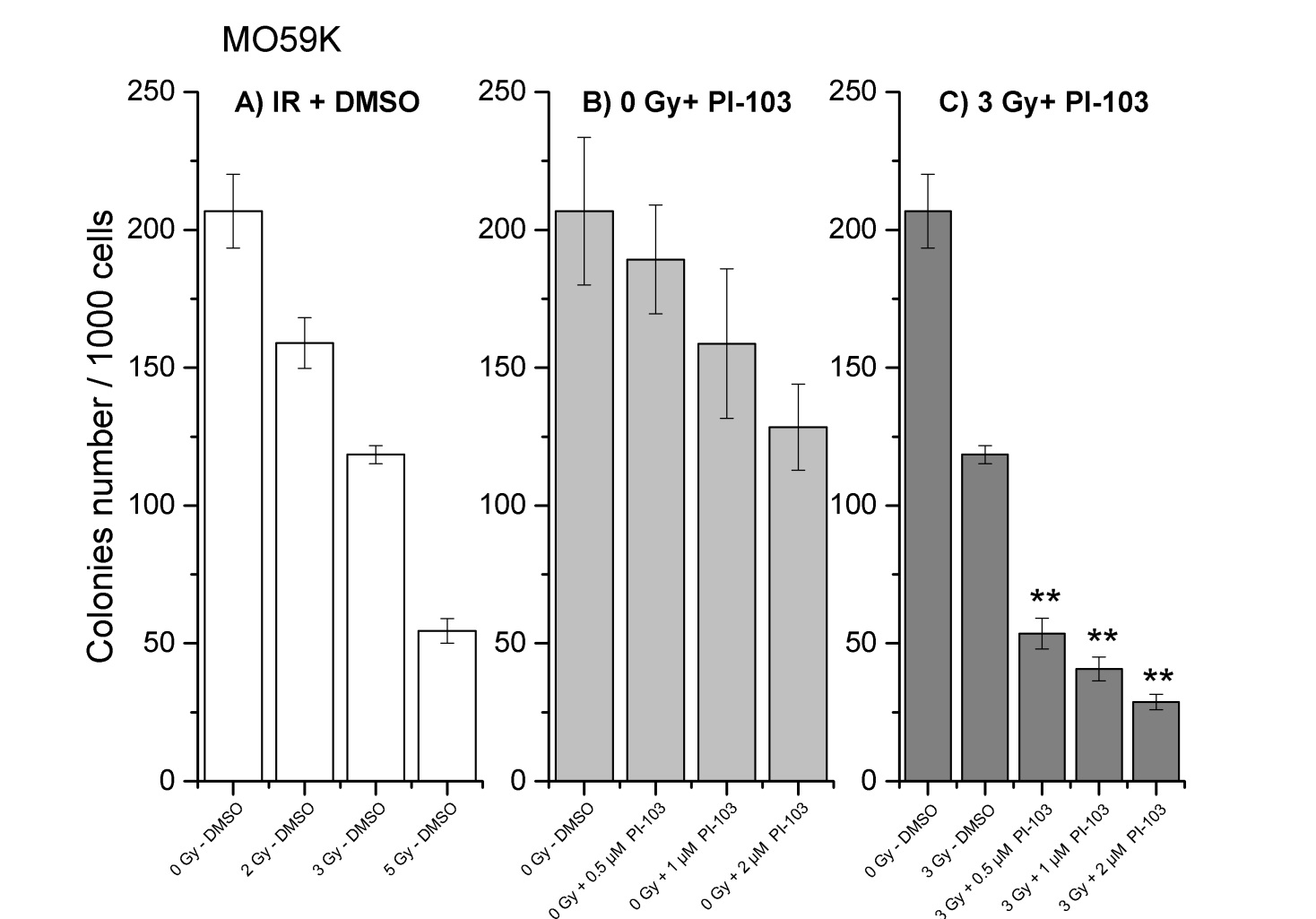 |
| --- |
| **Figure S6.** Effects of radiation, PI-103 or combined PI-103-radiation treatments on colony-forming ability of MO59K cells. One thousand MO59K cells were plated on Petri dishes for 16 h, treated with radiation (**A**: 2, 3, and 5 Gy), or with PI-103 (**B**: 0.5 µM, 1 µM, and 2 µM) or with a combination of radiation (**C**: 3 Gy) and PI-103 (0.5 µM, 1 µM and 2 µM)) added 3 h before irradiation. Twenty four h post-irradiation the inhibitor was washed out and the cultures were incubated in fresh CGM for the next 12 days, fixed and stained with crystal violet. The numbers of colonies were counted and averaged from 3 independent experiments. The combinatory effect of radiation and PI-103 was synergistic in case of 1 µM and 2 µM PI-103 (the combination index was determined according Malyarenko et al. (2020) and Chou (2010) and found to be at both concentrations of PI-103 and radiation dose of 3 Gy < 0.7). |

| 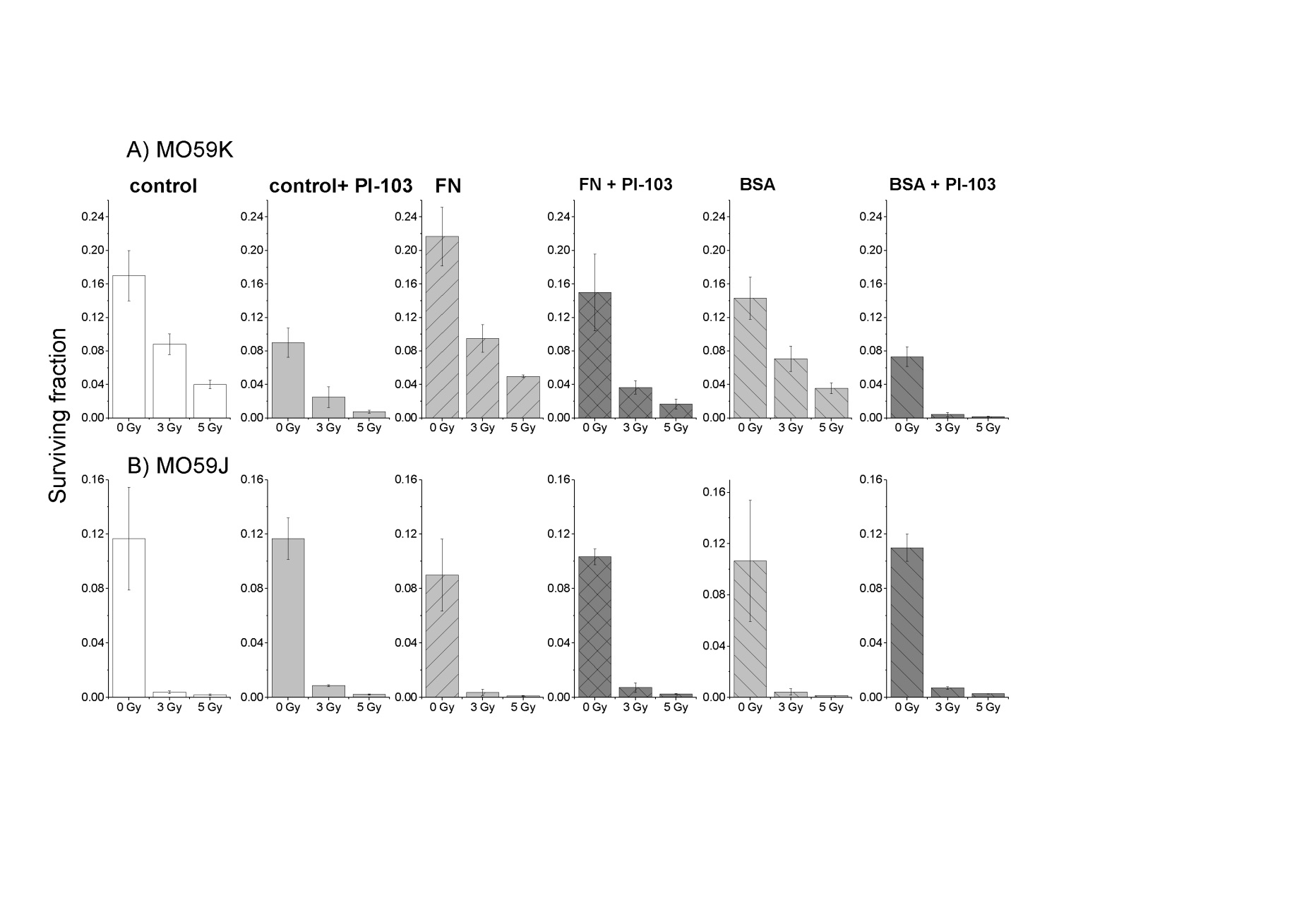 |
| --- |
| **Figure S7.**  MO59K (**A**) and MO59J (**B**) cells were plated either on polystyrene (control, uncoated), fibronectin or BSA and exposed to PI-103, irradiation or combined drug-IR treatments, and the clonogenic survival was determined. |

| 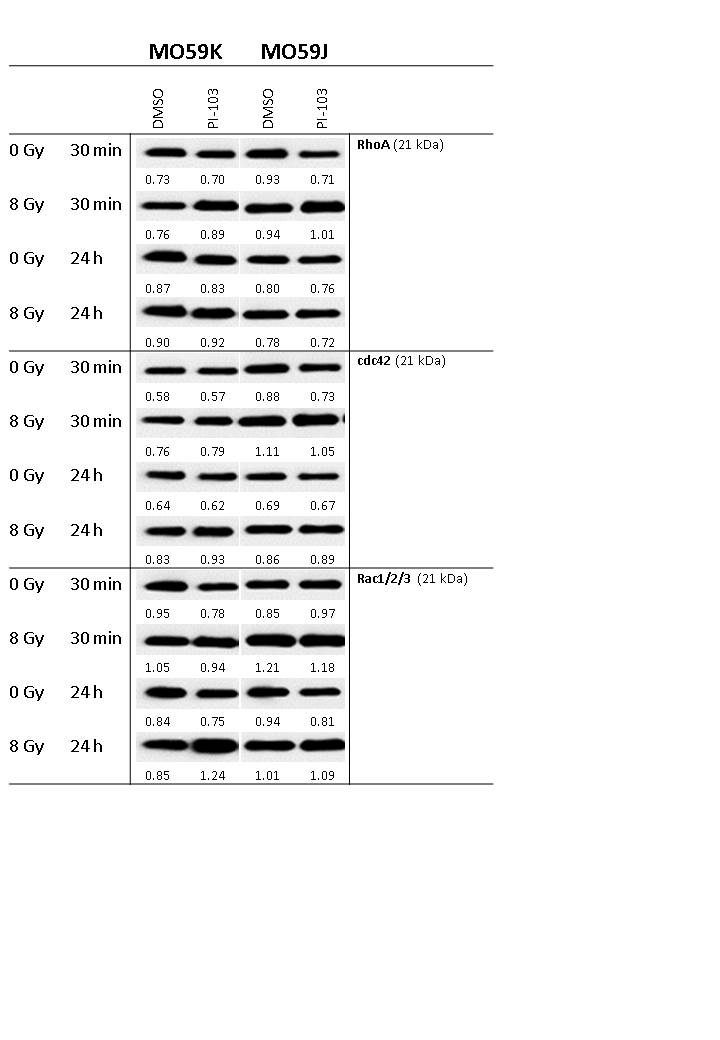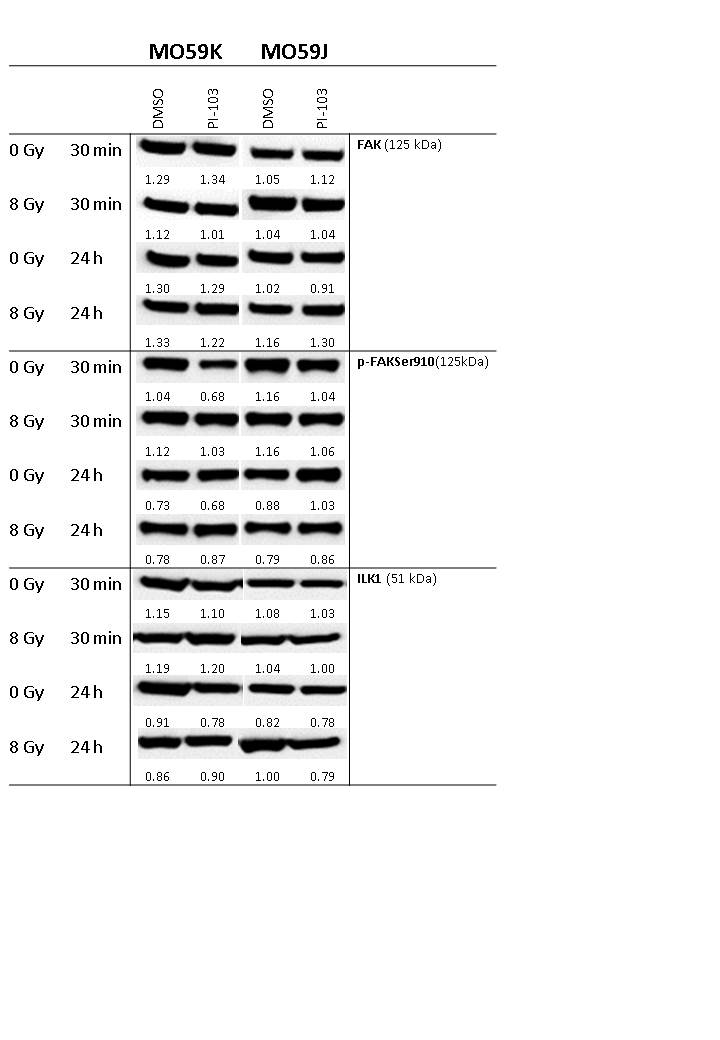 |
| --- |
| **Figure S8.** Representative Western blot analysis of expression levels of several adhesion-associated proteins in MO59K and MO59J tumor cells treated with DMSO (control) or PI-103 for 3 h prior to IR with 8 Gy and detected 30 min and 24 h thereafter. β-actin was used as loading control. The experiment was repeated at least three times. For details, *see* legend to Figure 2. |

| 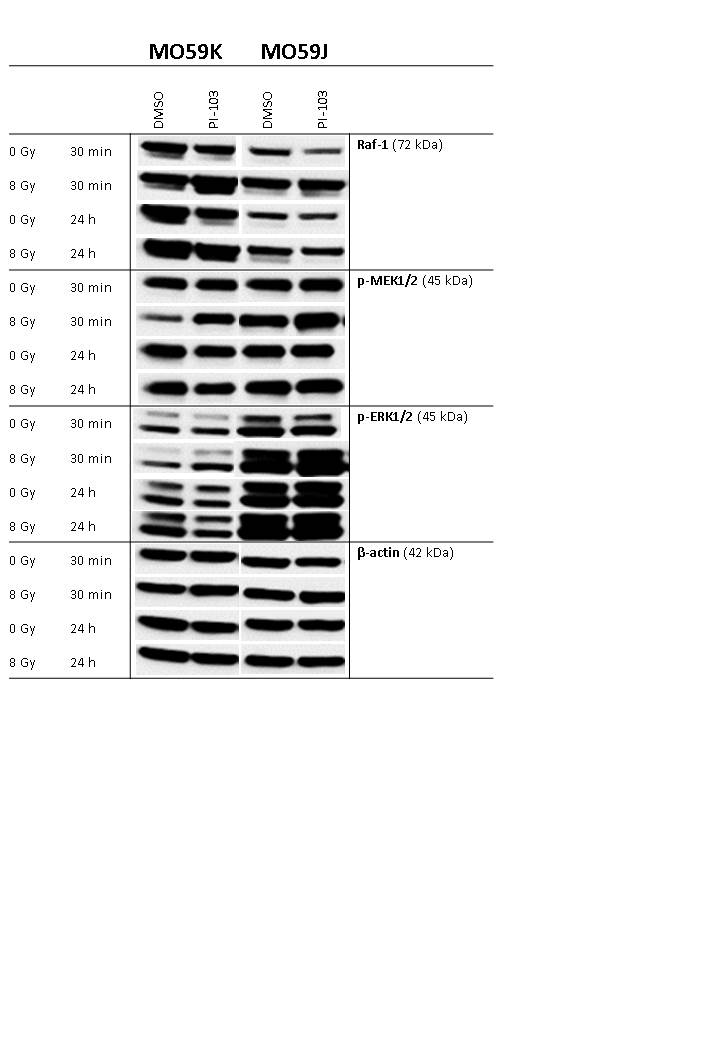 |
| --- |
| **Figure S9.** Representative Western blot analysis of expression levels of several marker proteins of MAPK-pathway in MO59K and MO59J tumor cells treated with DMSO (control) or PI-103 for 3 h prior to IR with 8 Gy and detected 30 min and 24 h thereafter. The uncropped blots are shown in Fig. S12. β-actin was used as loading control. The experiment was repeated at least three times. For details, *see* legend to Figure 2. |

| 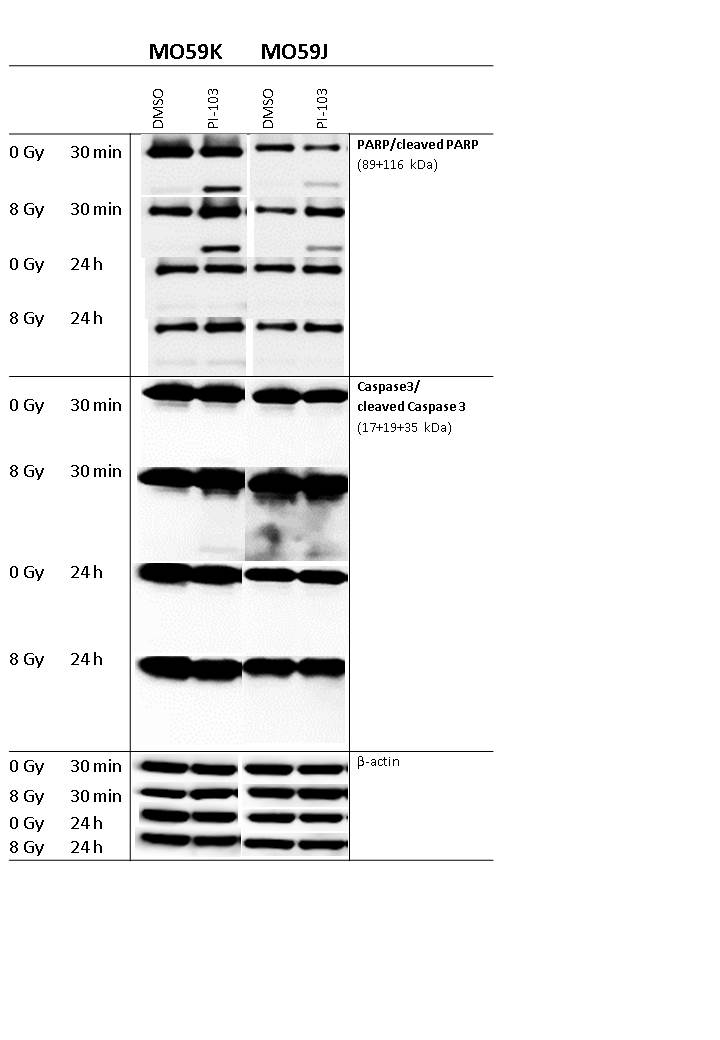 |
| --- |
| **Figure S10.** Western blot analysis of PARP and cleaved PARP proteins in MO59K and MO59J cells treated with DMSO (control) or PI-103 for 3 h prior to IR with 8 Gy and detected 30 min and 24 h thereafter. The uncropped blots are shown in Fig. S12. β-actin was used as loading control. The experiments were repeated at least three times. For details, *see* legend to Figure 2. |

| 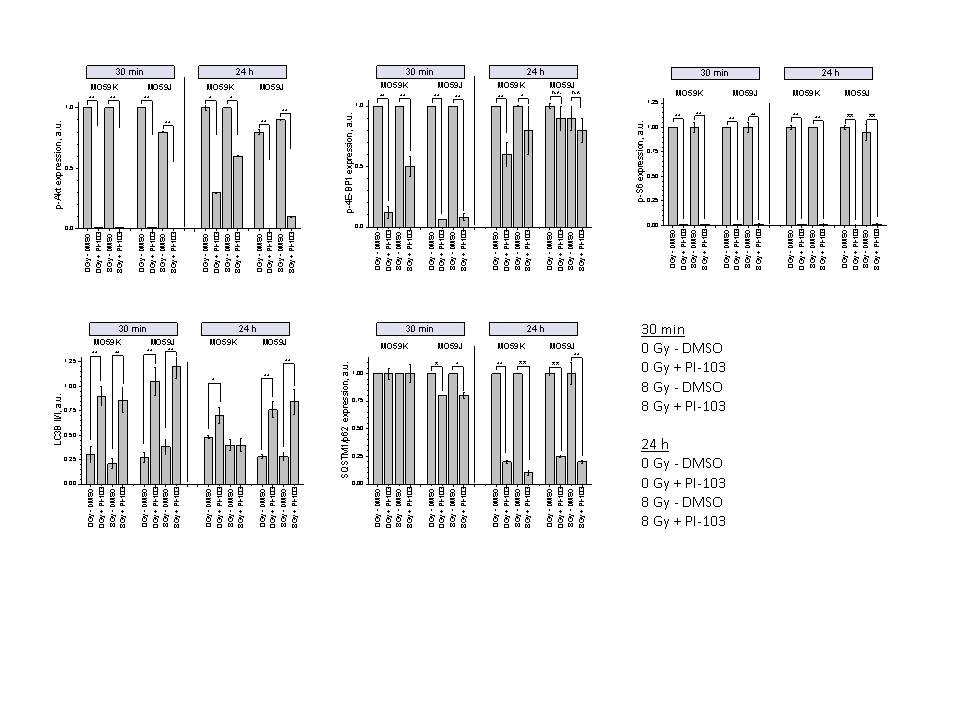 |
| --- |
| **Figure S11.** Normalized expression of marker proteins (p-Akt, p-4E-BP1, p-S6, LC3B II/I and SQSTM1/p62) in MO59K and MO59J cells treated with DMSO (control) or PI-103 for 3 h prior to IR with 8 Gy and detected 30 min and 24 h thereafter. The experiments were repeated at least three times, mean values of protein content ± SE are shown. Differences between PI-103 and/or IR treatment were statistically analyzed using Student’s *t*-test. The mean value of protein content in control non-irradiated samples was set to 1. For details, *see* legend to Figure 2. |

## Table S1

Cloning efficiencies and radiosensitivity parameters**^a^** of *in vitro* irradiated tumor cell lines untreated and pretreated (3 h) prior to IR with the PI-103 and re-plated 24 h post-IR

| **Cell line** | **Plating efficiency** | **SF2 ^b^** |  | **D_10_ (Gy)^c^** |  | **IF_10_^d^**  **(D_10_ control)/**  **(D_10_+inh.)** |  |
| --- | --- | --- | --- | --- | --- | --- | --- |
| **MO59K** – contr. | 0.15±0.04 | 0.55±0.04 | **_*_** | 6.1±0.4 | **_*_** | 1.0 | **_*_** |
| PI-103 | 0.17±0.07 | 0.43±0.04 |  | 4.8±0.2 |  | 1.3±0.1 |  |
| **MO59J** – contr. | 0.20±0.07 | 0.14±0.02 | **_*_** | 2.3±0.15 | **_*_** | 1.0 | **_*_** |
| PI-103 | 0.21±0.08 | 0.24±0.04 |  | 3.2±0.16 |  | 0.7±0.1 |  |

**^a^**Mean (± SE) from at least three independent experiments;

**^b^**SF2 is the colony-forming ability at 2 Gy;

**^c^**D_10_ is the radiation dose required to reduce colony-forming ability by 10%;

**^d^**The growth inhibition factor IF_10_ was calculated as (D_10_ control)/(D_10_+inh.);

**^*^**means statistical significance of the differences at *P* < 0.05.
