# Supplementary material for: Opposite effects of the triple target (DNA-PK/PI3K/mTOR) inhibitor PI-103 on the radiation sensitivity of glioblastoma cell lines proficient and deficient in DNA-PKcs
Source: BMC Cancer. 2021 Nov 11;21:1201. doi: 10.1186/s12885-021-08930-1 (PMC8582108; doi:10.1186/s12885-021-08930-1)
Supplement: Supplementary file 2 — Additional file 2: Supplementary Fig. S12. Uncropped representative Western blots for Figs. 2-6, and Supplementary Figs. S8-S10. The figures show original blots, in some cases the same membrane was used to simultaneously detect different proteins (after cutting) which strongly deviated in molecular weight. All original blots for β-actin used as loading control for the respective runs are also included. [file 12885_2021_8930_MOESM2_ESM.docx]

**Supplementary Information:**

**Uncropped Western blots corresponding to Figures 2, 4-7 and Figures S8-S10**

**PI3K (Fig. 2)**

**** **MO59K MO59J**


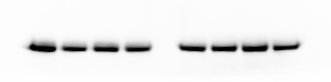
30 min 0 Gy


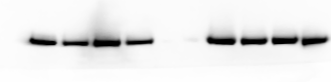
30 min 8 Gy


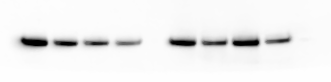
24 h 0 Gy


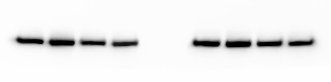
24 h 8 Gy

**The uncropped blots of protein PI3K for Figure 2**. Representative Western blot analysis of expression levels of several marker proteins of PI3K-pathway in MO59K and MO59J tumor cells treated with DMSO (control) or the indicated inhibitors (PI-103 and NVP-AUY922, alone or in a combination) for 3 h prior to IR with 8 Gy and detected 30 min and 24 h thereafter. The experiment was repeated at least four times.

**p-Akt (Fig. 2)**

**** **MO59K MO59J**


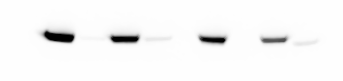
 30 min 0 Gy


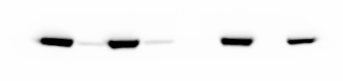
 30 min 8 Gy


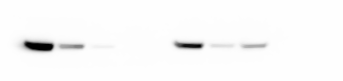
 24 h 0 Gy


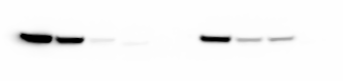
 24 h 8 Gy

**The uncropped blots of p-Akt protein for Figure 2**. Representative Western blot analysis of expression levels of several marker proteins of PI3K-pathway in MO59K and MO59J tumor cells treated with DMSO (control) or the indicated inhibitors (PI-103 and NVP-AUY922, alone or in a combination) for 3 h prior to IR with 8 Gy and detected 30 min and 24 h thereafter. The experiment was repeated at least four times.

**mTOR (Fig. 2)**

**MO59K MO59J**


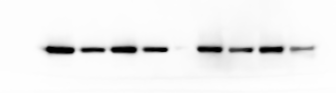
30 min 0 Gy


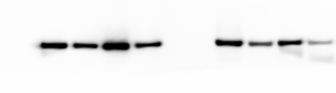
30 min 8 Gy


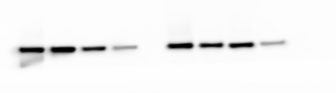
24 h 0 Gy


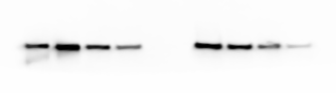
24 h 8 Gy

**The uncropped blots of mTOR protein for Figure 2.** Representative Western blot analysis of expression levels of several marker proteins of PI3K-pathway in MO59K and MO59J tumor cells treated with DMSO (control) or the indicated inhibitors (PI-103 and NVP-AUY922, alone or in a combination) for 3 h prior to IR with 8 Gy and detected 30 min and 24 h thereafter. The experiment was repeated at least four times.

**p-4E-BP1 (Fig. 2)**

 **MO59K MO59J**


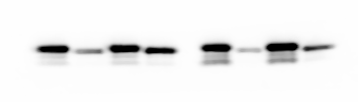
30 min 0 Gy


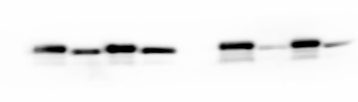
30 min 8 Gy


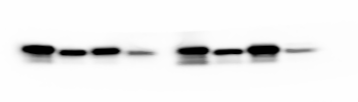
24 h 0 Gy


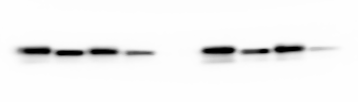
24 h 8 Gy

**The uncropped blots of p-4E-BP1 protein for Figure 2**. Representative Western blot analysis of expression levels of several marker proteins of PI3K-pathway in MO59K and MO59J tumor cells treated with DMSO (control) or the indicated inhibitors (PI-103 and NVP-AUY922, alone or in a combination) for 3 h prior to IR with 8 Gy and detected 30 min and 24 h thereafter. The experiment was repeated at least four times.

**p-S6 (Fig. 2)**

 **MO59K MO59J**


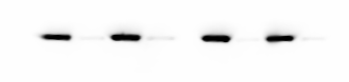
30 min 0 Gy


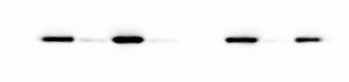
30 min 8 Gy


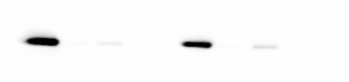
24 h 0Gy


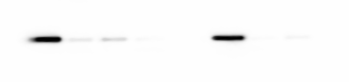
24 h 8 Gy

**The uncropped blots of p-S6 protein for Figure 2.** Representative Western blot analysis of expression levels of several marker proteins of PI3K-pathway in MO59K and MO59J tumor cells treated with DMSO (control) or the indicated inhibitors (PI-103 and NVP-AUY922, alone or in a combination) for 3 h prior to IR with 8 Gy and detected 30 min and 24 h thereafter. The experiment was repeated at least four times.

**β-actin (Fig. 2)**

**** **MO59K MO59J**


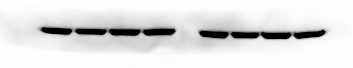
30 min 0 Gy


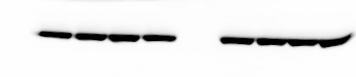
30 min 8 Gy


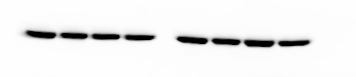
24 h 0 Gy


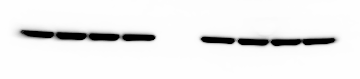
24 h 8 Gy

**The uncropped blots of β-actin for Figure 2.** Representative Western blot analysis of expression levels of β-actin used as loading control in MO59K and MO59J tumor cells treated with DMSO (control) or the indicated inhibitors (PI-103 and NVP-AUY922, alone or in a combination) for 3 h prior to IR with 8 Gy and detected 30 min and 24 h thereafter. The experiment was repeated at least four times.

**Ku70 (Fig. 4)**

**MO59K MO59J**


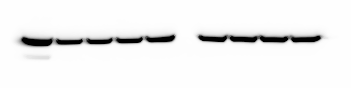
30 min

0 Gy 8 Gy 0 Gy 8 Gy
 C PI C PI C PI C PI


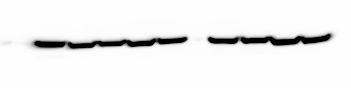
24 h

0 Gy 8 Gy 0 Gy 8 Gy
 C PI C PI C PI C PI

**The uncropped blots of Ku70 protein for Figure 4**. Representative Western blot analysis of expression levels of DNA repair proteins belonging to NHEJ pathway in MO59K and MO59J tumor cells treated with DMSO (control) or the inhibitor PI-103 for 3 h prior to IR with 8 Gy and detected 30 min and 24 h thereafter. The experiment was repeated at least four times.

(“C” denotes *Control, DMSO treatment*; “PI” denotes PI-103).

**Ku80 (Fig. 4)**

**MO59K MO59J**


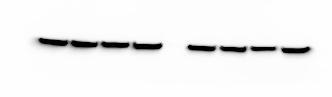
 30 min

0 Gy 8 Gy 0 Gy 8 Gy
 C PI C PI C PI C PI


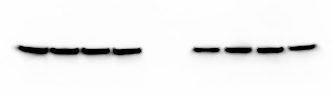
 24 h

0 Gy 8 Gy 0 Gy 8 Gy
 C PI C PI C PI C PI

**The uncropped blots of Ku80 protein for Figure 4.** Representative Western blot analysis of expression levels of DNA repair proteins belonging to NHEJ pathway in MO59K and MO59J tumor cells treated with DMSO (control) or the inhibitor PI-103 for 3 h prior to IR with 8 Gy and detected 30 min and 24 h thereafter. The experiment was repeated at least four times.

(“C” denotes *Control, DMSO treatment*; “PI” denotes PI-103).

**DNA-PKcs (Fig. 4)**

 **MO59K MO59J**


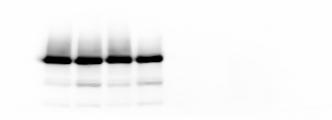
30 min 0 Gy


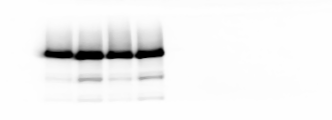
30 min 8 Gy


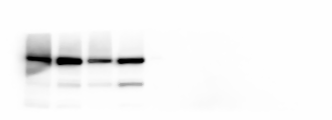
24 h 0 Gy


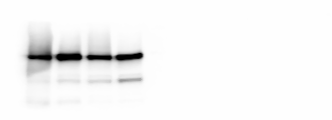
24 h 8 Gy

**The uncropped blots of DNA-PKcs for Figure 4**. Representative Western blot analysis of expression levels of DNA repair proteins belonging to NHEJ pathway in MO59K and MO59J tumor cells treated with DMSO (control) or the indicated inhibitors (PI-103 and NVP-AUY922, alone or in a combination) for 3 h prior to IR with 8 Gy and detected 30 min and 24 h thereafter. The experiment was repeated at least four times.

Note that the MO59J cells do not express DNA-PKcs.

**Rad50 (Fig. 4)**

**MO59K MO59J**

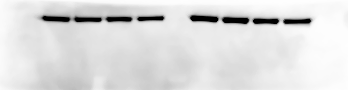
30 min 0 Gy


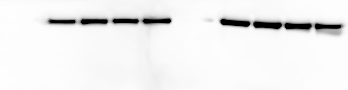
30 min 8 Gy


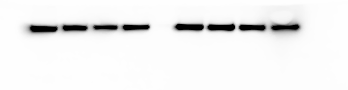
24 h 0 Gy


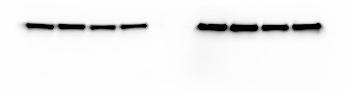
24 h 8 Gy

**The uncropped blots of Rad50 protein for Figure 4**. Representative Western blot analysis of expression levels of DNA repair proteins belonging to NHEJ pathway in MO59K and MO59J tumor cells treated with DMSO (control) or the indicated inhibitors (PI-103 and NVP-AUY922, alone or in a combination) for 3 h prior to IR with 8 Gy and detected 30 min and 24 h thereafter. The experiment was repeated at least four times.

**ß-actin (Fig. 4)**

**** **MO59K MO59J**


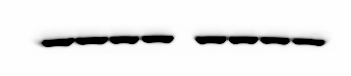
30 min 0 Gy


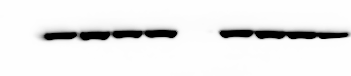
30 min 8 Gy


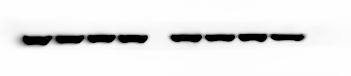
24 h 0 Gy


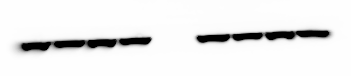
24 h 8 Gy

**The uncropped blots of ß-actin for Figure 4**. Representative Western blot analysis of expression levels of DNA repair proteins belonging to NHEJ pathway in MO59K and MO59J tumor cells treated with DMSO (control) or the indicated inhibitors (PI-103 and NVP-AUY922, alone or in a combination) for 3 h prior to IR with 8 Gy and detected 30 min and 24 h thereafter. The experiment was repeated at least four times.

**ATM (Fig. 5)**

**** **MO59K MO59J**


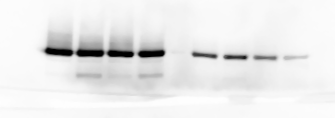
 30 min 0 Gy


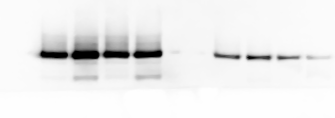
 30 min 8 Gy


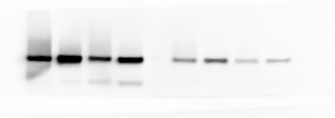
24 h 0 Gy


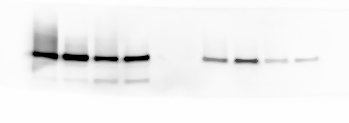
 24 h 8 Gy

**The uncropped blots of ATM protein for Figure 5.** Representative Western blot analysis of expression levels of DNA repair proteins belonging to HR pathway in MO59K and MO59J tumor cells treated with DMSO (control) or the indicated inhibitors (PI-103 and NVP-AUY922, alone or in a combination) for 3 h prior to IR with 8 Gy and detected 30 min and 24 h thereafter. The experiment was repeated at least four times.

**CtIP (Fig. 5)**

**MO59K MO59J**


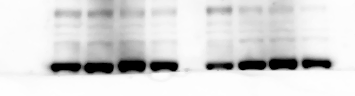
30 min

0 Gy 8 Gy 0 Gy 8 Gy
 C PI C PI C PI C PI


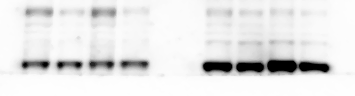
24 h

0 Gy 8 Gy 0 Gy 8 Gy
 C PI C PI C PI C PI

**The uncropped blots of CtIP protein for Figure 5.** Representative Western blot analysis of expression levels of DNA repair proteins belonging to HR pathway in MO59K and MO59J tumor cells treated with DMSO (control) or the inhibitor PI-103 for 3 h prior to IR with 8 Gy and detected 30 min and 24 h thereafter. The experiment was repeated at least four times.

(“C” denotes *Control, DMSO treatment*; “PI” denotes PI-103).

**p95/NBS1 (Fig. 5)**

**** **MO59K MO59J**


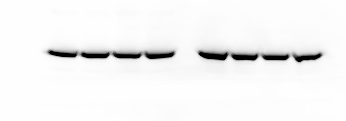
30 min 0 Gy


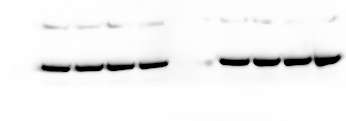
30 min 8 Gy


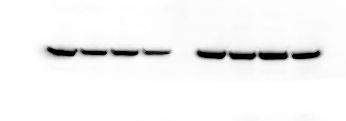
24 h 0 Gy


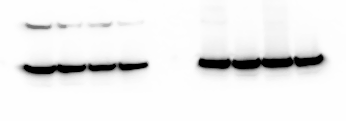
24 h 8 Gy

**The uncropped blots of p95/NBS1 protein for Figure 5.** Representative Western blot analysis of expression levels of DNA repair proteins belonging to NHEJ pathway in MO59K and MO59J tumor cells treated with DMSO (control) or the indicated inhibitors (PI-103 and NVP-AUY922, alone or in a combination) for 3 h prior to IR with 8 Gy and detected 30 min and 24 h thereafter. The experiment was repeated at least four times.

**p-BRCA1 (Fig. 5)**

**MO59K MO59J**


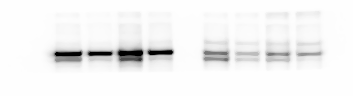
30 min 0 Gy - 8 Gy

0 Gy 8 Gy 0 Gy 8 Gy
 C PI C PI C PI C PI


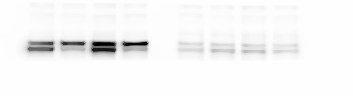
24 h 0 Gy - 8 Gy

0 Gy 8 Gy 0 Gy 8 Gy
 C PI C PI C PI C PI

**The uncropped blots of p-BRCA1 protein for Figure 5**. Representative Western blot analysis of expression levels of DNA repair proteins belonging to NHEJ pathway in MO59K and MO59J tumor cells treated with DMSO (control) or the inhibitor PI-103 for 3 h prior to IR with 8 Gy and detected 30 min and 24 h thereafter. The experiment was repeated at least four times.

(“C” denotes *Control, DMSO treatment*; “PI” denotes PI-103).

**Rad51 (Fig. 5)**

**** **MO59K MO59J**


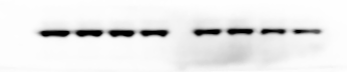
30 min 0 Gy


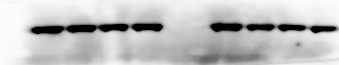
30 min 8 Gy


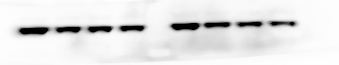
24 h 0 Gy


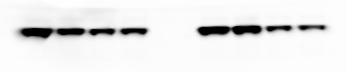
24 h 8 Gy

**The uncropped blots of Rad51 protein for Figure 5**. Representative Western blot analysis of expression levels of DNA repair proteins belonging to NHEJ pathway in MO59K and MO59J tumor cells treated with DMSO (control) or the inhibitor PI-103 for 3 h prior to IR with 8 Gy and detected 30 min and 24 h thereafter. The experiment was repeated at least four times.

(“C” denotes *Control, DMSO treatment*; “PI” denotes PI-103).

**Rad54 (Fig. 5)**

**** **MO59K MO59J**


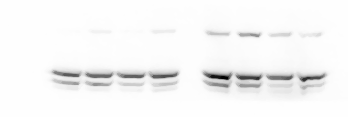
 30 min 0 Gy


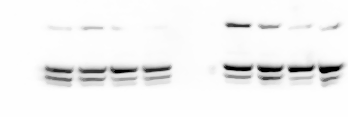
 30 min 8 Gy


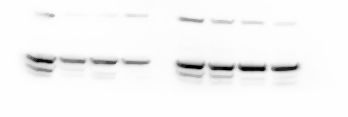
 24 h 0 Gy


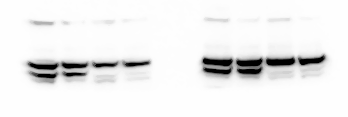
 24 h 8 Gy

**The uncropped blots of Rad54 protein for Figure 5**. Representative Western blot analysis of expression levels of DNA repair proteins belonging to NHEJ pathway in MO59K and MO59J tumor cells treated with DMSO (control) or the indicated inhibitors (PI-103 and NVP-AUY922, alone or in a combination) for 3 h prior to IR with 8 Gy and detected 30 min and 24 h thereafter. The experiment was repeated at least four times.

**ß-actin (Fig. 5)**

**** **MO59K MO59J**


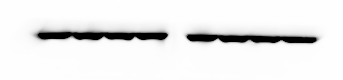
30 min 0 Gy


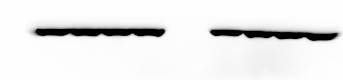
30 min 8 Gy


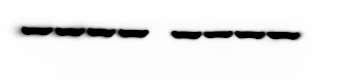
24 h 0 Gy


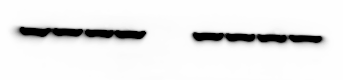
24 h 8 Gy

**The uncropped blots of ß-actin for Figure 5.** Representative Western blot analysis of expression levels of β-actin used as loading control in MO59K and MO59J tumor cells treated with DMSO (control) or the indicated inhibitors (PI-103 and NVP-AUY922, alone or in a combination) for 3 h prior to IR with 8 Gy and detected 30 min and 24 h thereafter. The experiment was repeated at least four times.

**p53, p-p53 (Fig. 6)**

**MO59K MO59J**


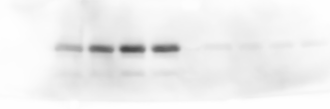
30 min

0 Gy 8 Gy 0 Gy 8 Gy
 C PI C PI C PI C PI


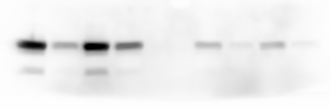
24 h

0 Gy 8 Gy 0 Gy 8 Gy
 C PI C PI C PI C PI

**The uncropped blots of p53 protein for Figure 6.**


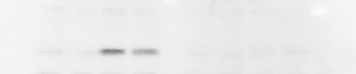
30 min

0 Gy 8 Gy 0 Gy 8 Gy
 C PI C PI C PI C PI


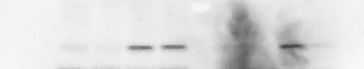
24 h

0 Gy 8 Gy 0 Gy 8 Gy
 C PI C PI C PI C PI

**The uncropped blots of p-p53 protein for Supplementary Figure 6.**(“C” denotes *Control, DMSO treatment*; “PI” denotes PI-103).

**Bax (Fig. 6)**

**MO59K MO59J**


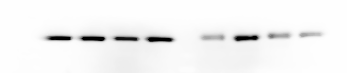
30 min

0 Gy 8 Gy 0 Gy 8 Gy
 C PI C PI C PI C PI


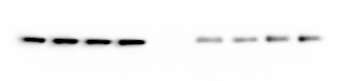
24 h

0 Gy 8 Gy 0 Gy 8 Gy
 C PI C PI C PI C PI

**The uncropped blots of Bax protein for Supplementary Figure 6.** Representative Western blot analysis of expression levels of p53, p-p52 and Bax proteins in MO59K and MO59J tumor cells treated with DMSO (C - control) or the indicated inhibitor (PI denotes PI-103) for 3 h prior to IR with 8 Gy and detected 30 min and 24 h thereafter. The experiment was repeated at least four times.

**LC3B (Fig. 7)**

**** **MO59K MO59J**


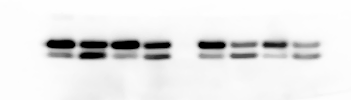
 30 min 0 Gy


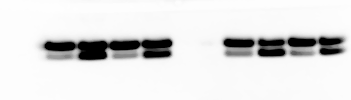
 30 min 8 Gy


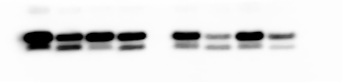
 24 h 0 Gy


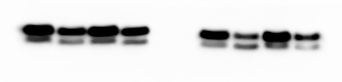
 24 h 8 Gy

**The uncropped blots of LC3B proteins for Figure 7.** Representative Western blot analysis of expression levels of LC3B proteins in MO59K and MO59J tumor cells treated with DMSO (control) or the indicated inhibitors (PI-103 and NVP-AUY922, alone or in a combination) for 3 h prior to IR with 8 Gy and detected 30 min and 24 h thereafter. The experiment was repeated at least four times.

**SQSTM1/p62 (Fig. 7)**

**** **MO59K MO59J**


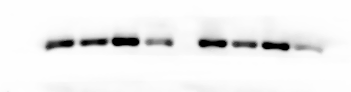
 30 min 0 Gy


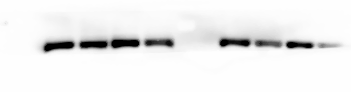
 30 min 8 Gy


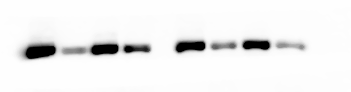
 24 h 0 Gy


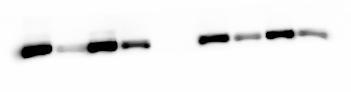
 24 h 8 Gy

**The uncropped blots of SQSTM1/p62 protein for Figure 7.** Representative Western blot analysis of expression levels of SQSTM1/p62 protein in MO59K and MO59J tumor cells treated with DMSO (control) or the indicated inhibitors (PI-103 and NVP-AUY922, alone or in a combination) for 3 h prior to IR with 8 Gy and detected 30 min and 24 h thereafter. The experiment was repeated at least four times.

**ß-actin (Fig. 7)**

**** **MO59K MO59J**


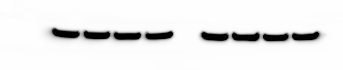
30 min 0 Gy


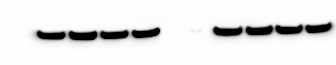
30 min 8 Gy


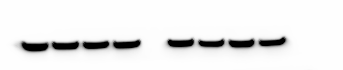
24 h 0Gy


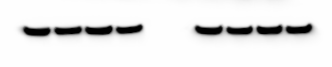
24 h 8 Gy

**The uncropped blots of ß-actin for Figure 7.** Representative Western blot analysis of expression levels of ß-actin protein used as loading control in MO59K and MO59J tumor cells treated with DMSO (control) or the indicated inhibitors (PI-103 and NVP-AUY922, alone or in a combination) for 3 h prior to IR with 8 Gy and detected 30 min and 24 h thereafter. The experiment was repeated at least four times.

**Supplement**

**FAK (Fig. S8)**

**** **MO59K MO59J**


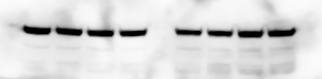
30 min 0 Gy


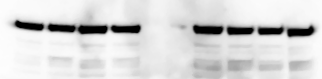
30 min 8 Gy


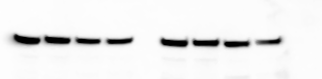
24 h 0 Gy


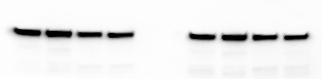
24 h 8 Gy

**The uncropped blots of FAK protein for Supplementary Figure S8.** Representative Western blot analysis of expression levels of adhesion-associated protein FAK in MO59K and MO59J tumor cells treated with DMSO (control) or the indicated inhibitors (PI-103 and NVP-AUY922, alone or in a combination) for 3 h prior to IR with 8 Gy and detected 30 min and 24 h thereafter. The experiment was repeated at least four times.

**p-FAKSer910 (Fig. S8)**

**** **MO59K MO59J**


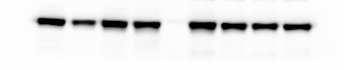
30 min 0 Gy


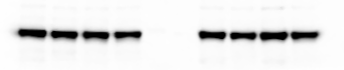
30 min 8 Gy


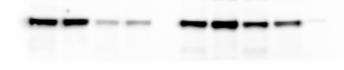
24 h 0 Gy


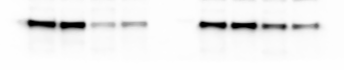
24 h 8 Gy

**The uncropped blots of p-FAKSer910 protein for Supplementary Figure S8.** Representative Western blot analysis of expression levels of adhesion-associated protein p-FAKSer910 in MO59K and MO59J tumor cells treated with DMSO (control) or the indicated inhibitors (PI-103 and NVP-AUY922, alone or in a combination) for 3 h prior to IR with 8 Gy and detected 30 min and 24 h thereafter. The experiment was repeated at least four times.

**ILK1 (Fig. S8)**

**MO59K MO59J**


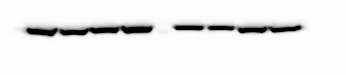
 30 min

0 Gy 8 Gy 0 Gy 8 Gy
 C PI C PI C PI C PI


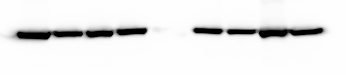
 24 h

0 Gy 8 Gy 0 Gy 8 Gy
 C PI C PI C PI C PI

**The uncropped blots of ILK1 protein for Supplementary Figure S8.** Representative Western blot analysis of expression levels of adhesion-associated protein ILK1 in MO59K and MO59J tumor cells treated with DMSO (control) or the inhibitor PI-103 for 3 h prior to IR with 8 Gy and detected 30 min and 24 h thereafter. The experiment was repeated at least four times.

(“C” denotes *Control, DMSO treatment*; “PI” denotes PI-103).

**RhoA (Fig. S8)**

**** **MO59K MO59J**


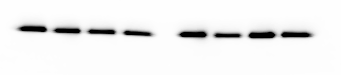
 30 min 0 Gy


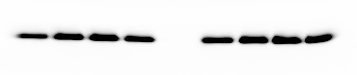
 30 min 8 Gy


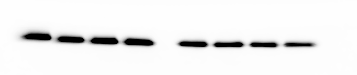
 24 h 0 Gy


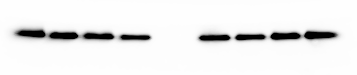
 24 h 8 Gy

**The uncropped blots of RhoA protein for Supplementary Figure S8.** Representative Western blot analysis of expression levels of adhesion-associated protein RhoA in MO59K and MO59J tumor cells treated with DMSO (control) or the indicated inhibitors (PI-103 and NVP-AUY922, alone or in a combination) for 3 h prior to IR with 8 Gy and detected 30 min and 24 h thereafter. The experiment was repeated at least four times.

**cdc42 (Fig. S8)**

**** **MO59K MO59J**


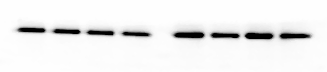
 30 min 0 Gy


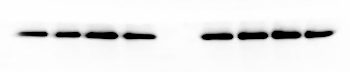
 30 min 8 Gy


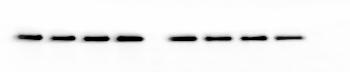
 24 h 0 Gy


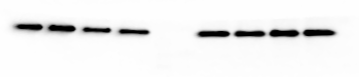
 24 h 8 Gy

**The uncropped blots of cdc42 protein for Supplementary Figure S8.** Representative Western blot analysis of expression levels of adhesion-associated protein cdc42 in MO59K and MO59J tumor cells treated with DMSO (control) or the indicated inhibitors (PI-103 and NVP-AUY922, alone or in a combination) for 3 h prior to IR with 8 Gy and detected 30 min and 24 h thereafter. The experiment was repeated at least four times.

**Rac1/2/3 (Fig. S8)**

**MO59K MO59J**

30 min 0 Gy

30 min 8 Gy

24 h 0 Gy

24 h 8 Gy

**The uncropped blots of Rac1/2/3 protein for Supplementary Figure S8.** Representative Western blot analysis of expression levels of adhesion-associated protein Rac1/2/3 in MO59K and MO59J tumor cells treated with DMSO (control) or the indicated inhibitors (PI-103 and NVP-AUY922, alone or in a combination) for 3 h prior to IR with 8 Gy and detected 30 min and 24 h thereafter. The experiment was repeated at least four times.

**ß-actin to p-FAKSer910 (Fig. S8)**

**MO59K MO59J**

30 min 0 Gy

30 min 8 Gy

24 h 0 Gy

24 h 8 Gy

**The uncropped blots of ß-actin for Supplementary Figure S8.** Representative Western blot analysis of expression levels of β-actin protein used a loading control in MO59K and MO59J tumor cells treated with DMSO (control) or the indicated inhibitors (PI-103 and NVP-AUY922, alone or in a combination) for 3 h prior to IR with 8 Gy and detected 30 min and 24 h thereafter. The experiment was repeated at least four times.

**ß-actin to ILK1 (Fig. S8)**

**MO59K MO59J**

30 min

0 Gy 8 Gy 0 Gy 8 Gy
 C PI C PI C PI C PI

24 h

0 Gy 8 Gy 0 Gy 8 Gy
 C PI C PI C PI C PI

**The uncropped blots of ß-actin for Supplementary Figure S8.** Representative Western blot analysis of expression levels of β-actin protein used a loading control in MO59K and MO59J tumor cells treated with DMSO (control) or the inhibitor PI-103 for 3 h prior to IR with 8 Gy and detected 30 min and 24 h thereafter. The experiment was repeated at least four times.

(“C” denotes *Control, DMSO treatment*; “PI” denotes PI-103).

**ß-actin to FAK, RhoA, cdc42, Rac1/2/3 (Fig. S8)**

**MO59K MO59J**

30 min 0 Gy

30 min 8 Gy

24 h 0 Gy

24 h 8 Gy

**The uncropped blots of ß-actin for Supplementary Figure S8.** Representative Western blot analysis of expression levels of β-actin protein used a loading control in MO59K and MO59J tumor cells treated with DMSO (control) or the indicated inhibitors (PI-103 and NVP-AUY922, alone or in a combination) for 3 h prior to IR with 8 Gy and detected 30 min and 24 h thereafter. The experiment was repeated at least four times.

**Raf-1 (Fig. S9)**

**MO59K MO59J**

30 min 0 Gy

30 min 8 Gy

24 h 0 Gy

24 h 8 Gy

**The uncropped blots of Raf-1 protein for Supplementary Figure S9.** Representative Western blot analysis of expression levels of Raf-1 protein in MO59K and MO59J tumor cells treated with DMSO (control) or the indicated inhibitors (PI-103 and NVP-AUY922, alone or in a combination) for 3 h prior to IR with 8 Gy and detected 30 min and 24 h thereafter. The experiment was repeated at least four times.

**p-MEK1/2 (Fig. S9)**

**MO59K MO59J**

30 min 0 Gy

30 min 8 Gy

24 h 0 Gy

24 h 8 Gy

**The uncropped blots of p-MEK1/2 protein for Supplementary Figure S9.** Representative Western blot analysis of expression levels of p-MEK1/2 protein in MO59K and MO59J tumor cells treated with DMSO (control) or the indicated inhibitors (PI-103 and NVP-AUY922, alone or in a combination) for 3 h prior to IR with 8 Gy and detected 30 min and 24 h thereafter. The experiment was repeated at least four times.

**p-ERK1/2 (Fig. S9)**

**MO59K MO59J**

30 min 0 Gy

30 min 8 Gy

24 h 0 Gy

24 h 8 Gy

**The uncropped blots of p-ERK1/2 protein for Supplementary Figure S9.** Representative Western blot analysis of expression levels of p-ERK1/2 protein in MO59K and MO59J tumor cells treated with DMSO (control) or the indicated inhibitors (PI-103 and NVP-AUY922, alone or in a combination) for 3 h prior to IR with 8 Gy and detected 30 min and 24 h thereafter. The experiment was repeated at least four times.

**ß-actin (Fig. S9)**

**MO59K MO59J**

30 min 0 Gy

30 min 8 Gy

24 h 0 Gy

24 h 8 Gy

**The uncropped blots of ß-actin for Supplementary Figure S9.** Representative Western blot analysis of expression levels of β-actin protein used a loading control in MO59K and MO59J tumor cells treated with DMSO (control) or the indicated inhibitors (PI-103 and NVP-AUY922, alone or in a combination) for 3 h prior to IR with 8 Gy and detected 30 min and 24 h thereafter. The experiment was repeated at least four times.

**PARP, cleaved PARP (Fig. S10)**

**MO59K MO59J**

30 min 0 Gy

30 min 8 Gy

24 h 0Gy

24 h 8 Gy

**The uncropped blots of PARP and cleaved PARP proteins for Supplementary Figure S10.** Representative Western blot analysis of expression levels of PARP and cleaved PARP proteins in MO59K and MO59J tumor cells treated with DMSO or the indicated inhibitors (PI-103 and NVP-AUY922, alone or in a combination) for 3 h prior to IR with 8 Gy and detected 30 min and 24 h thereafter. The experiment was repeated at least four times.

**Caspase 3, cleaved Caspase 3 (Fig. S10)**

**MO59K MO59J**

30 min 0 Gy

30 min 8 Gy

24 h 0 Gy

24 h 8 Gy

**The uncropped blots of Caspase 3 and cleaved Caspase 3 for Supplementary Figure S10.** Representative Western blot analysis of expression levels of caspase 3 and cleaved caspase 3 in MO59K and MO59J tumor cells treated with DMSO (control) or the indicated inhibitors (PI-103 and NVP-AUY922, alone or in a combination) for 3 h prior to IR with 8 Gy and detected 30 min and 24 h thereafter. The experiment was repeated at least four times.

**ß-actin (Fig. S10)**

**MO59K MO59J**

30 min 0 Gy

30 min 8 Gy

24 h 0 Gy

24 h 8 Gy

**The uncropped blots of ß-actin for Supplementary Figure S10.** Representative Western blot analysis of expression levels of β-actin used as loading control in MO59K and MO59J tumor cells treated with DMSO (control) or the indicated inhibitors (PI-103 and NVP-AUY922, alone or in a combination) for 3 h prior to IR with 8 Gy and detected 30 min and 24 h thereafter. The experiment was repeated at least four times.
